# Humanitarian management strategy for interstate movement of migrant workers in India during COVID-19 pandemic: an optimization based approach

**DOI:** 10.1007/s10479-023-05199-4

**Published:** 2023-02-14

**Authors:** Niladri Palit, Atanu Chaudhuri, Nishikant Mishra

**Affiliations:** 1 Queen’s University Belfast, Belfast, UK; 2 Durham University, Durham, UK; 3 University of Hull, Hull, UK

**Keywords:** Economic impact of COVID-19, Optimization in humanitarian crisis, Migrant worker movement in India

## Abstract

India faced a unique situation during the ongoing COVID-19 pandemic when millions of migrant workers, in different states had to be transported to their home states as workplaces shut down. The governments in respective states faced challenges of minimizing economic impact while ensuring that the risk of infection was also kept under control. This paper develops models based on various secondary data from governmental and relevant non-governmental sources, trying to minimize the economic impact while keeping the rate of infection low and determining whether the migrant workforce should be allowed to stay in their workplace state or allowed to return to their home state. We found that the number of days of lockdown had a significant impact on the results. Fewer days of lockdown resulted in workers remaining in their work state as the preferred outcome, while a higher number of days of lockdown implied that people traveled to their home state and remain there. The proportion of workers who were willing to return to their work state played an important role on the results too. Beyond the threshold percentages of migrant workers returning to their work state, it became optimal for the government to encourage the workers to travel to their home state. However, this was mostly visible for moderate number of lockdown days as the effects on results were dominated by the impact from the number of lockdown days for too high or too low number of lockdown days. There is also an important trade-off between the budget and infection rate ‘R’ for the governments to consider. Minimizing the risk of infection requires an additional budget.

## Introduction

In India, a large section of the population migrates to different states with higher economic activity than their home states in search of jobs. Based on the census data of 2011,^1^ nearly 37% of the entire population of the country was considered as migrant population for various reasons (Singh, 2020b; MoHA, 2021). The railways’ data shows that the average flow of migration due to work-related reasons between 2011 and 2016 was 9 million per year. (Jadhav, 2021). Maji et al. (2020) proposed some models for forecasting the population data in their work, based on the previous census data. From their calculation, some of the notable migrant worker movements took place, a combined impact of influx and outflux, in states such as Maharashtra (7.1%), Delhi NCR (21.0%), Karnataka (8.8 %), Haryana (1.9%), Uttar Pradesh (9.2%), and Bihar (13.4%). This migrant population plays a key role in contributing to the economic activity of the states they travel to for work. It was found that 70% of migrant workers are primary school dropouts. This has left them with the option to become daily wage workers. Unemployment avoidance was cited by 83% of the respondents as the principal reason for their migration (United Nations, 2021). Compounding this, the COVID-19 related lockdown and closures of factories or construction activities led many of this migrant workforce to return to their native states to be with their family and avoid paying rent. In May 2020, the Government of India and various State Governments arranged special trains and buses for the stranded migrant workers (Jain, 2020a). However, the spread of COVID-19 is positively associated with the infected patient’s travel history (Kraemer et al., 2020; Chinazzi et al., 2020) while limited people’s movement enables low-level community transmission of COVID-19 (Shammi et al., 2021).

Research suggests that the human infection risk can be high due to the length of the exposure time window, transmission routes and structural characteristics during travel or work. This can result in the rapid spread of the infection (Shen et al., 2020). Furthermore, Husain and Kothari (2021) highlighted the generation of new Covid hotspots due to such movement compounded by infrastructural challenges in the states where the migrant workers traveled. Hence, repatriating migrant workers could increase the risk of spreading COVID-19 within the communities in their native places. This leads to the question about an alternative approach or strategy to repatriate them to their state of domicile. One such strategy is to allow the workers to stay in the states where they were working prior to lockdown. On the contrary, Rahaman et al. (2021) conducted a study on migrant workers stranded in the states where they were working prior to the COVID-19 outbreak. The authors argued that poor housing conditions, and lack of hygiene along with co-morbid health conditions, put these worker communities at risk of COVID-19 and its spread. Keeping migrant workers in their respective work locations involved the costs of hosting them in relief centers or at their own homes while the movement of the workers was expected to increase the rate of infection in the destination states. Hence, it was difficult for the Government to decide which strategy would be the most effective in minimizing the risk of the spread of COVID-19 while also minimizing cost. It has become apparent that the tools of risk analysis are important (Hass, 2020) as they provide analytical support to decision makers. While there are studies on the impact of lockdown on COVID-19 transmission in India (Chatterjee et al., 2020; Mukhopadhyay & Chakraborty, 2020) and estimating the transportation requirements (Maji et al., 2020), there is no research to date on determining whether the migrant workers should stay in their work locations or return to their home states while trying to optimize the economic impact of such decisions. Hence, we aim to address the research question: *What could be the strategy (sending the migrant workers back to their state of domicile or making arrangements in their work state) to effectively manage this precarious humanitarian situation of stranded migrant workers considering the overall economic impact as well as the risk of spreading the COVID-19?* This will be answered through the specific objectives

- To determine the number of migrant workers who could be transported from their working state to their home state and brought back after the lockdown, and the number of days for the transportation operation while optimizing the overall economic impact.
- To determine the number of migrant workers who could remain at their workplace state while minimizing the economic impact and the rate of infection.

The key contributions of this research are modeling a realistic policy decision regarding the transportation of migrant workers in India while considering both the economic and infection risk, demonstrating the usefulness of data-driven decision making by collecting relevant data, which governments can use to take such decisions, analyzing trade-offs, and developing recommendations based on threshold values of percentage of workers willing to return in addition to the days of lockdown. The rest of this paper is structured as follows: literature review, problem description and model formulation, numerical analysis, implications of the results and finally the conclusion.

## Literature review

The humanitarian crisis and its management involving operations and supply chain issues are not new concepts. This attracted several research papers including literature reviews (de Camargo Fiorini et al., 2021; Wamba, 2020; Modgil et al., 2020; Behl & Dutta, 2019), the application of data analytics (Dubey et al., 2019) and some of the behavioural issues such as organizational culture (Altay et al., 2018) amongst many other themes. Despite many theoretical developments, humanitarian organizations often face challenges to manage their disaster management operations effectively and efficiently (Dubey et al., 2020). The pandemic of COVID-19 led to a humanitarian disaster with a significant impact across the world. This section details some related developments in the literature and finally identifies the research gaps that we addressed through this research.

Gaskin et al. (2021) found that the number of deaths and cases of COVID -19 were positively correlated with the number of airports, train stations, and the percentage of adults using public transportation. Thus, the population living in cities with multiple railway stations and airports were at a high risk of infection and death. Sangiorgio and Parisi (2020) developed an index to predict the risk of contagion in urban districts to support administrations in identifying the best strategies to reduce or restart local activities during lockdown conditions. The economic and social effects of the COVID-19 outbreak on public transportation extended beyond service performance and health risks to financial viability, social equity, and sustainable mobility (Tirachini & Cats, 2020).

One of the key cornerstone academic developments of pandemic risk management is susceptible-infected-recovered (SIR) and susceptible-exposed-infectious-recovered (SEIR) models. Kermack and McKendrick (1927) proposed the SIR model to examine the spread of contagious diseases in a homogeneous population. The SEIR model was used to study the seasonal pattern of varicella epidemics in France (Deguen et al., 2000).

Demirci et al. (2011) formulated and analysed a fractional SEIR epidemic model to determine a disease-free equilibrium, and a non-stability condition for positive equilibrium. The proposed model helped decision-makers to determine a disease-free state when the death rate was proportional to the population density. During the epidemic, the reproduction number was the most common indicator for understanding and determining the contagiousness of an infection. Multiple methods have been used by authors to estimate the transmission risk of COVID-19 and its implication such as the statistical exponential growth model (Li et al., 2020), statistical maximum likelihood estimation (Liu et al., 2020), Stochastic Markov Chain Monte Carlo methods (Wu et al., 2020), the Stochastic simulations method (Riou & Althaus, 2020) and vector regression model (Parbat & Chakraborty, 2020). The SIR epidemiology model was used to determine the optimal lockdown policy (duration and strictness of lockdown measures), and to minimize the number of fatalities and lockdown costs (Alvarez et al., 2020). Bhar and Malliaris (2021) used the Markov switching econometric model to estimate the impact of COVID-19’s unconventional monetary policy and compared it to the monetary policy during the global financial crisis in the USA. By contrast, in the absence of a COVID-19 vaccine, Ferguson et al. (2020) presented a microsimulation model to study the impact of suppression and mitigation measures on monitoring the spread of COVID-19 in the UK and the USA. Vrabac et al. (2021) obtained the effects of transportation on the Spread of COVID-19 with a Multi-Networked SEIR Model. Qian and Ukkusuri (2021) developed the Trans-SEIR model to understand the transmission of infectious diseases through travel and activity contagion in urban areas. Wan et al. (2020) examined the impact of community and social mobilization on COVID-19 pandemic management in Hong Kong. Atkeson (2020), Stock (2020), and Acemoglu et al. (2020) used the SIR model to predict the spread of COVID-19 in the United States and calculated its impact on the economy. The main goal was to determine the optimal policy by making a trade-off between efforts needed to save lives and improving economic indicators.

There are multiple studies with many research agendas, which used the above epidemic models for decision support for COVID-19. Maji et al. (2020) investigated the potential surge in confirmed and active cases of COVID-19 infection using a modified SEIR model and determined the train and bus fleet size required for repatriating migrant workers. Other studies have explored various socio-cultural and transport measures during COVID-19 (Shortall et al., 2022). Issues associated with socio-cultural or demographical challenges related to this migrant movement in India during COVID-19 were also addressed (Carswell et al., 2022). Some authors further argued that an absence of adequate health screening at both the source and destination, over-crowded trains, insanitary conditions, and scheduling problems with the trains led to the creation of hotspots of COVID-19 due to interstate travel (Husain & Kothari, 2021). However, there is somewhat limited knowledge from these studies about possible alternative approaches. Thus, the question that remains is what would have been an alternate strategic choice for the policymakers to better manage this migrant movement situation.

The economic objective is one of the most important cornerstones of disaster management and humanitarian operations that has attracted many academic publications (Turkeš et al., 2019). Zhang et al. (2019) proposed mathematical models in their work to access the economic impacts. Galindo and Batta (2013) in their review of Operations Research models for disaster operations management noted that there were virtually no articles related to business continuity or infrastructure design. Some recent studies by Goldschmidt and Kumar (2019) proposed contradictory views with the findings of limited to no evidence of a better cost performance with disaster management preparedness. Despite these, there was a strong claim by the United Nations to emphasize the importance of disaster readiness leading to improved future investment decisions (Goldschmidt & Kumar, 2019). In the context of COVID-19, as different governments across the world grappled to minimize the rate of infection rates and death among its population, there also had to be an assurance of minimum disruption to economic activity. In the context of India, as noted in the introduction, COVID-19 related lockdown measures led to migrant workers moving to their state of domicile from the workplace states before the pandemic. The following were researched: the general state of the art of the crisis (Suresh et al., 2020; Misra & Gupta, 2021), human rights (Kumar & Choudhury, 2021), risk of spread of infection due to planning issues (Pal et al., 2021), implications for transportation (Maji et al., 2020), social and health implications (Iyengar & Jain, 2021; Jesline et al., 2021; Choudhari, 2020), and the role of non-profit organisations (Barhate et al., 2021) among others. The economic implications of the migrant worker movement due to COVID-19 have received somewhat limited attention. Although authors Misra and Gupta (2021) noted the importance of the implications of these migrant workers for economic activities, the objective was mostly restricted to identifying different themes using data mining. Other authors investigated the economic performances from a fiscal policy-making perspective (Thakur & Kumar, 2021; Jose et al., 2020). The findings of Goswami et al. (2021) highlight the need for more robust economic strategies for recovery. Summarizing these findings, it can be said that in the context of the decision to transport migrant workers in India, the economic objective along with the constraint to keep the infection rate under control should be considered. However, there are very limited studies, which addressed the conundrum of whether the migrant workers should be sent back to their home state or accommodated in relief facilities in the workplace state, or asked to stay in their homes in their workplace state with further government support for daily maintenance while minimizing the economic impact and the risk of spread of infection. This research gap leads to the question of a strategic choice of addressing the key trade-off between controlling the spread of the pandemic (by controlling R value) against minimizing the economic impact due to this exodus migrant movement. Thus, we strive to address the overarching aim noted in the introduction in Sect. 1.

## Problem description and model formulation

**Fig. 1:**
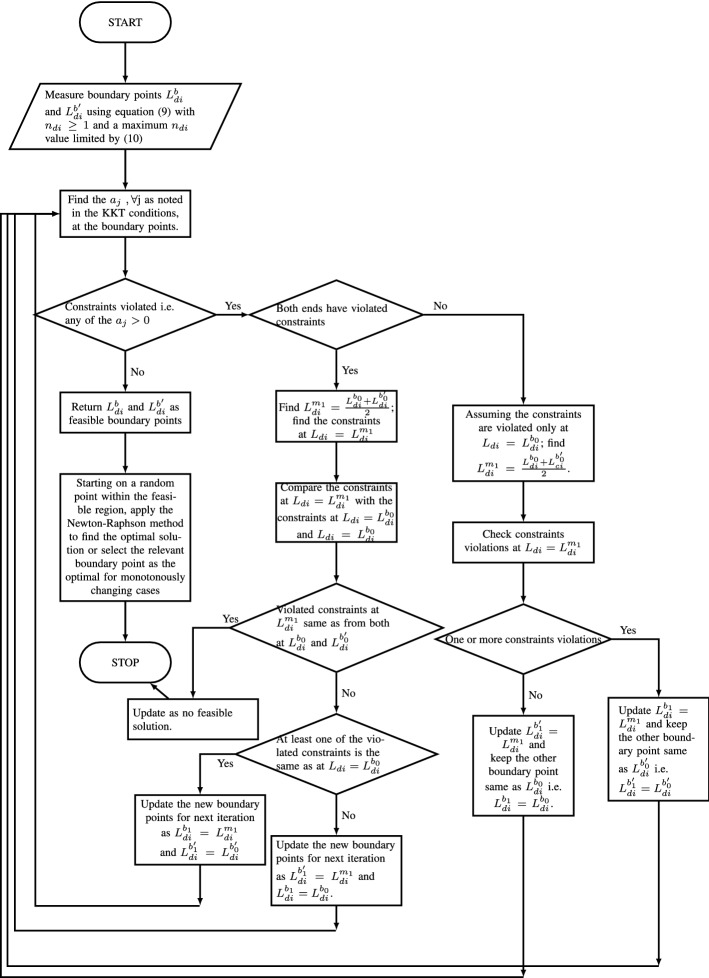
Search heuristic using mid-points between the boundary points

Following the limitations highlighted in the literature review in Sect. 2, this section highlights the problem under consideration in words, then mathematical terms. On 24th March 2020, a nationwide lockdown was announced in India to control the spread of the COVID-19 virus. Due to workplaces having to close their operations temporarily, many unskilled to semi-skilled workers lost their daily jobs temporarily. This left them with limited or no choices but to travel to their home state to avoid financial difficulties in their then current residence state prior to lockdown. As mentioned in the introduction, we examined two types of strategies in this paper to manage this humanitarian crisis situation: migrant workers travelling to their home state from their resident state where they were working prior to lockdown or staying in their resident state. As per reports from the 2011 census, approximately 37% of the country’s population was considered as migrant workers in some capacity (Singh, 2020b). According to Singh (2020b) and Sharma (2020), there are some economically developed states where there has been significant migration over the last few years. This was corroborated in the research of Barhate et al. (2021), where the authors plotted a map denoting the migrant movement during the COVID-19 crisis in early 2020 (See fig. 1, page 153). Earlier in the introduction section, we presented some migration statistics noted by Maji et al. (2020) which offered insights into the states that witnessed the highest percentage of worker movement. Considering only the influx of migrant workers to a particular state based on the statistics presented, the key states with the highest migration were Delhi NCR, Maharashtra, and Karnataka. Taking this into consideration, we examined these three states Maharashtra, Karnataka, and Delhi NCR for this research. At first, we noted the list of acronyms used in the model. We followed it up with the assumptions and the model formulations and solution methodologies for the two different cases.

### List of acronyms

The list of acronyms used in this research is noted in Tables 1 and 2.

**Table 1:** Objective function and decision variables

$E_{ei}$ or $E_{si}$:	Total cost/monetary impact to state i’s economy
$L_{di}:$	Number of state i migrant workers who can be transported for the departure case per day
$L_{ri}$:	Number of migrant workers to state i who can be transported for the return case per day
$L_{hi}$:	Number of workers to be asked to stay in their residence
$L_{ci}$:	Number of people who can be accommodated in relief camp facilities
$n_{di}$:	Number of days the migration movement can be run for the departure from state i
$n_{ri}$:	Number of days the migration movement can be run for the return to state i

**Table 2:** Model parameters

$L_{i}$:	Total number of migrant workers in state i
$p_{ri}$:	Proportion of the overall worker population likely to return if transported back to home state
$p_{li}$:	Proportion of the overall worker population likely to require government help with a settlement
	if they remain in state they were working or travel to their home state
$p_{ini}$:	Proportion of people likely to be infected in state i
$R_i$:	Rate of infection in state i
$N_{ti}$:	Number of trains that can be run considering network constraints in state i
$N_{mi}$:	Number of health inspections that can be done by an ICMR approved centers per day in any state i
$N_{hi}$:	Maximum number of hospital beds that can be arranged in a day in state i
$N_{ci}$:	Maximum number of people that can be accommodated in each relief camp following with UNHCR safety guidelines
$M_i$:	Number of medical centers available per day in state i
$\rho _{ci}$:	Number of relief camps that can be set up in state i
$T_{di}$:	Capacity of each train considering safe social distancing
$T_{ri}$:	Capacity for reverse migration transport
$n_{hi}$:	Number of days treated in a hospital if a COVID-19 tested worker is required to be admitted in a hospital in state i
$n_{li}$:	Total number of days of lockdown in state i
$C_{ti}$:	Cost per running a train in state i
$C_{mi}$:	Cost of testing medical safety before allowing to board onto the train in state i per unit worker
$$C_{ci} $$:	Cost per person in a relief camp per day excluding food in state i
$$C_{fi} $$:	Cost of food per person in a relief camp per day in state i
$C_{si}$:	Subsistence to be paid to each worker staying in his/her own accommodation per day in state i
$C_{hi}$:	Cost of hospital bed per patient per day in state i
$B_{i}$:	Available transport budget for state i
$G_i$:	State of working’s GDP contribution per worker
$T_e$:	Average latency period
$T_g$:	Generation period
x:	Proportion of people exposed
y:	Proportion of the number of days of lockdown to allow the dpearture transportation operation to continue
Y(t):	Number of infected people by time t

### Initial assumptions

To conduct a numerical analysis, we assumed the following

- All the migrant workers have no symptomatic cases of COVID-19 infection and/or all migrant workers would be allowed to board the trains after the essential medical examinations if they are tested negative.
- Each migrant worker had equal productivity contributing to the gross domestic product of the economy
- The migrant workers needed to be paid the minimum daily wage rate equivalent if they were asked to stay at home.
- Anyone requiring hospital admission would be treated in 15 days. This assumption was relaxed further to analyze the cases where people admitted to hospitals required more than 15 days of treatment.

### The models and solution methodologies

The key aim of this study is to examine the strategy (keeping the migrant workers where they were working prior to the pandemic or transporting them to their state of domicile) that could be adopted to manage the economic impact, and the spread of the pandemic. This in turn converts to an optimization problem of minimizing the economic impact (in terms of cost or expenditure) that is constrained by various resourcing and capacity issues, and the need for tighter control over the spread of the pandemic. The following steps were followed in addressing the optimization problem mentioned above.

*Step 1* We defined the objective functions for the two cases.

* Case I* The migrant workers being transported to their home state

* Case II* The migrant workers staying in the state where they work

* Step 2* Identify all the variables that would be contributing towards the objective function ($L_{di}$, $L_{ri}$, $n_{di}$, and $n_{ri}$ in Case I, and $L_{ci}$ and $L_{hi}$ for Case II)

* Step 3* Identify all the constraints for the two cases

* Step 4* Identify all the parameters from the constraints and the objective functions from both the cases

* Step 5* Collect data from various secondary sources. To solve the optimization problem, we needed to collect data from various secondary sources such as governmental databases, newspaper publications, and UNHCR.

* Step 6* Initialize all the model parameters based on the collected data for both cases.

*Step 7* Solve and analyze the models numerically

*Step 8* Generate the results and present them.

*Step 9* We changed the model parameters to examine how the models will work through some further sensitivity analysis.

#### Case I—migrant workers being transported to their home state

Indian Railways operated special trains across various places in India, which were called ”Shramik Special” to allow migrant workers to travel from their state of residence to their home state after the lockdown started (Special Correspondent, 2020b). The objective of the proposed model is to minimize the overall economic impact. This included the economic loss due to: the workers those who stayed back in their state of residence and were not working during the lockdown [(the first component in Eq. (1)], the workers who traveled to their home state and were expected to return post lockdown [(the second component in Eq. (1)], and the workers travelling to their home state, but who were not expected to return [(the third component in Eq. (1)]. We assumed that the migrant daily workers contributed every day prior 25th March, 2020. The rest of the 281 working days would be lost from the contribution to that state’s economy for those who did not return after they traveled to their home state. Furthermore, we added the costs to operate the “Shramik Special" trains (including the return), the cost incurred to conduct the tests for the workers before they were allowed to board the trains, and the cost incurred to treat the workers who were infected with COVID-19. Due to the sudden emergent nature of the humanitarian crisis, there were limited options for conducting rigorous medical tests for the migrant workers before they were allowed to board onto the mode of transportation. This could lead to the rapid spread of the virus due to the migrant workers travelling in confined places for a long time with somewhat limited opportunities to social distance (Maji et al., 2020). Thus, we proposed that the migrant workers would be tested for COVID-19 before they were allowed to onboard the train.

The proposed model is subjected to constraints. The number of people were sent to their state of domicile from where they were working before lockdown and brought back once the lockdown was over were constrained by the capacity of each train (following safety guidelines of social distancing in the case of sending back). Dividing the capacity by the number of people on board determined the number of trains required. Some of the workers who tested positive due to infection did not travel and might have required hospital treatment. However, we designed the constraint to calculate the number of trains required considering the maximum number of workers ($L_{di}$) traveling. This designed the model from a worst-case scenario. This was constrained by the number of trains that could be run per day as noted in constraints (2) and (3). Dividing the number of workers to be sent back by the number of ICMR approved tests/medical centers would yield the capacity requirement of each test center in the respective states. This was constrained by the maximum current capacity of the medical centers as noted in constraint (4). The left-hand side of constraint (5) represents the total number of people likely to be hospitalized which is constrained by the maximum number of hospital beds that can be arranged or are available. Assuming P as the number of people likely to be hospitalized i.e. $P=(p_{ini}*L_{di})*(1+R_i)$, we modeled acknowledging that all the P number of people infected with COVID-19 would require hospitalization for the duration of $n_{hi}$. This allowed us to model the situation against the worst-case scenario. We further assume a planning duration equal to the number of lockdown days, namely of $n_{li}$. For the duration of $n_{hi}$, the number of hospital beds available is $(N_{hi}-P)$. For rest of the $(n_{li}-n_{hi})$ duration, the number of beds available should be the $N_{hi}$. Hence, the weighted average availability of hospital beds became $\frac{(N_{hi}-P)(n_{hi})+(n_{li}-n_{hi})(N_{hi})}{n_{li}}$. Thus, the constraint should be $P \le \frac{(N_{hi}-P)(n_{hi})+(n_{li}-n_{hi})(N_{hi})}{n_{li}}$. Rearranging this and replacing P, we got the constraint noted in (5). The R value considered in (6) was based on the calculations provided by Fang et al. (2020) following the SEIR model. The $\lambda$ is considered as $\frac{\ln (Y(t))}{t}$ (Fang et al., 2020). For case I, we assumed that the government would only allow a maximum of $L_{di}$ number of migrant workers to make arrangements for travel each day as a protective measure to curb the spread of infection of COVID-19 and ask the remainder to be wait in their respective homes for their turn. Hence, it could be assumed $L_{di}$ number of people would be exposed and susceptible to the infection. Thus, we considered $Y(t)= p_{ini}*n_{di}*L_{di}$. The government aimed to restrict the R value to a maximum of 1 following the standard global guidelines. To conduct the operation, the government incurred expenses. Regarding constraint (7), the left-hand side had the following expenditures: The total cost to run the trains to transport the workers to their state of domicile and bring them back (derived by multiplying the number of trains to be run per day by the total number of days and cost to run each train), the cost incurred to conduct the medical examination before sending the migrant workers to their state of domicile, and the cost incurred to treat the infected workers. The total of these expenditures was constrained by the budget restrictions for the relief operation. Furthermore, we had constraints noting that: the sum total number of migrant workers requiring government help and those who did not require, should be equal to the total migrant workers in the relevant state (Eq. 8); the total number of migrant workers to return post lockdown should be equal to the proportion returned out of the total transported to the state of domicile (Eq. 9); the total number of days of the operation to transport workers to their state of domicile was restricted by a certain proportion of the number of days of lockdown [(y in this case was obtained by Eq. (10)]. This was to ensure that this activity was not prolonged, leading to a spread of the infection and uncertainty and confusion among people about whether they would be able to travel to their home or not. Considering the strict requirements of social distancing, the capacity of each train was less than under normal circumstances. Thus, it was expected that the number of days required to send the workers back would be either equal or more than the number of days required to bring them back after the lockdown restrictions had been eased. This was captured in Eq. (11). We further made a practical assumption to set them equal later on. The non-negativity constraints (Eq. 12). The model is presented below.

1 $$\begin{aligned} \min : E_{ei}&= \left[ \{(L_i-n_{di}*L_{di})*n_{li}+L_{ri}*n_{ri}*(n_{li}+n_{ri})\right. \nonumber \\&\quad \left. +(L_{di}*n_{di}-L_{ri}*n_{ri})*281\}*G_i\right] \nonumber \\&\quad +\left[ \left( \frac{n_{di}*L_{di}*C_{ti}}{T_{di}}\right) +\left( \frac{n_{ri}*L_{ri}*C_{ti}}{T_{ri}}\right) + \left( n_{di}*L_{di}*C_{mi}\right) \right] \nonumber \\&\quad +\left\{ p_{ini}*L_{di}*(1+R_i)*C_{hi}*n_{hi}\right\} \end{aligned}$$

S.t.

2 $$\begin{aligned}&\frac{L_{di}}{T_{di}} \le N_{ti} \end{aligned}$$

3 $$\begin{aligned}&\frac{L_{ri}}{T_{ri}} \le N_{ti} \end{aligned}$$

4 $$\begin{aligned}&\frac{L_{di}}{{N_{mi}}} \le M_i \end{aligned}$$

5 $$\begin{aligned}&(p_{ini}*L_{di}*n_{di})*(1+R_i) \le \frac{N_{hi}n_{li}}{(n_{li}+n_{hi})} \end{aligned}$$

6 $$\begin{aligned} R_i&=\left\{ (1+\lambda * T_e)*(1+\lambda *T_g)\right\} *x \le 1 \end{aligned}$$

7 $$\begin{aligned}&\quad \left( \frac{n_{di}*L_{di}*C_{ti}}{T_{di}}\right) +\left( \frac{n_{ri}*L_{ri}*C_{ti}}{T_{ri}}\right) \nonumber \\&\qquad \quad +\left( n_{di}*L_{di}*C_{mi}\right) +\left\{ p_{ini}*L_{di}*(1+R_i)*C_{hi}*n_{hi}\right\} \le B_{i} \end{aligned}$$

8 $$\begin{aligned}&L_{di}*n_{di}+L_{i}*(1-p_{li}) =L_i \end{aligned}$$

9 $$\begin{aligned}&p_{ri}* L_{di}*n_{di}= L_{ri}*n_{ri} \end{aligned}$$

10 $$\begin{aligned}&n_{di} \le y*n_{Li} \end{aligned}$$

11 $$\begin{aligned}&n_{di} \ge n_{ri} \end{aligned}$$

12 $$\begin{aligned}&L_{di}, L_{ri} \ge 0 \end{aligned}$$

This Case I migrant workers being transported to their state of domicile from the workplace states is a non-linear optimization problem. The objective function in Eq. (1), and the constraints (5), (6) and (7) are non-linear. This followed the definition of the R value from Fang et al. (2020) which has a logarithmic term of the decision variable of number of people likely to be infected as noted in the last paragraph. Thus, we tested the most commonly used techniques for assessing the optimality of non-linear cases using Karush Kuhn Tucker (KKT) conditions. If the conditions failed, then we needed to apply advanced search techniques to find out the near optimal solutions. To test the KKT conditions, we assumed the following

13 $$\begin{aligned} a_1&=\frac{(1-p_{ini})L_{di}}{T_{di}} - N_{ti} \end{aligned}$$

14 $$\begin{aligned} a_2&=\frac{L_{ri}}{T_{ri}}- N_{ti}\end{aligned}$$

15 $$\begin{aligned} a_3&=\frac{L_{di}}{N_{mi}}- M_{i}\end{aligned}$$

16 $$\begin{aligned} a_4&=(p_{ini}*L_{di}*n_{di})*(1+R_i) - \frac{N_{hi}n_{li}}{(n_{li}+n_{hi})}\end{aligned}$$

17 $$\begin{aligned} a_5&=\left( \frac{n_{di}*L_{di}*C_{ti}}{T_{di}}\right) +\left( \frac{n_{ri}*L_{ri}*C_{ti}}{T_{ri}}\right) + \left( n_{di}*L_{di}*C_{mi}\right) \end{aligned}$$

18 $$\begin{aligned}&\quad +\left\{ p_{ini}*L_{di}*n_{di}*(1+R_i)*C_{hi}*n_{hi}\right\} - B_i\end{aligned}$$

19 $$\begin{aligned} a_6&=\left\{ (1+\lambda * T_e)*(1+\lambda *T_g)\right\} *x - 1 \end{aligned}$$

20 $$\begin{aligned} a_7&= n_{di}-yn_{li} \end{aligned}$$

21 $$\begin{aligned} a_8&=n_{ri}-n_{di} \end{aligned}$$

22 $$\begin{aligned} b_1&=L_{di}*n_{di}+L_{i}*(1-p_{li})-L_i\end{aligned}$$

23 $$\begin{aligned} b_2&=p_{ri}* L_{di}*n_{di}- L_{ri}*n_{ri} \end{aligned}$$

Thus, the Lagrange multiplier function becomes

24 $$\begin{aligned}&\theta _{ei}= E_{ei}+\sum \lambda _j a_j+\sum \mu _k b_k \forall \, \text {j, k} \end{aligned}$$

25 $$\begin{aligned}&\textit{Optimality conditions for transporting case} \frac{\delta E_{ei}}{\delta V}+\sum \lambda _j \frac{\delta a_j}{\delta V}+\sum \mu _k \frac{\delta b_k}{\delta V}=0, \nonumber \\&\qquad , [\forall \, \text {j, k}], \, \text {V}\, \in \{L_{di}, L_{ri},n_{di}\} \end{aligned}$$

26 $$\begin{aligned}&\textit{Feasibility conditions for transporting case}\quad a_j+s_j=0 \, \, \text {and}\, \, b_k=0, \, \, [\forall \, \text {j, k}] \end{aligned}$$

27 $$\begin{aligned}&\textit{Complementary slackness conditions} s_j\lambda _j=0, \quad [\forall \, \text {j}] \end{aligned}$$

Using the optimality condition, we obtained the first optimality condition as

28 $$\begin{aligned} \begin{aligned}&{\lambda }_5\cdot \left( C_\text {hi}n_\text {d}n_\text {hi}p_\text {ini}\cdot \left( \dfrac{L_\text {di}\cdot \left( \frac{T_\text {e}\,\ln \left( p_\text {ini}L_\text {d}\right) }{n_\text {li}}+1\right) \left( \frac{T_\text {g}\ln \left( p_\text {ini}L_\text {d}\right) }{n_\text {li}}+1\right) }{L_\text {i}}+1\right) \right. \\&\left. +C_\text {hi}n_\text {d}n_\text {hi}p_\text {ini}L_\text {d}\cdot \left( \dfrac{\left( \frac{T_\text {e}\ln \left( p_\text {ini}L_\text {d}\right) }{n_\text {li}}+1\right) \left( \frac{T_\text {g}\ln \left( p_\text {ini}L_\text {d}\right) }{n_\text {li}}+1\right) }{L_\text {i}}\right. \right. \\&\left. \left. +\dfrac{T_\text {e}\cdot \left( \frac{T_\text {g}\ln \left( p_\text {ini}L_\text {d}\right) }{n_\text {li}}+1\right) }{L_\text {i}n_\text {li}}+\dfrac{T_\text {g}\cdot \left( \frac{T_\text {e}\ln \left( p_\text {ini}L_\text {d}\right) }{n_\text {li}}+1\right) }{L_\text {i}n_\text {li}}\right) +\dfrac{C_\text {ti}n_\text {d}}{T_\text {d}}+C_\text {mi}n_\text {d}\right) \\&+{\lambda }_4\cdot \left( n_\text {d}p_\text {ini}\cdot \left( \dfrac{L_\text {d}\cdot \left( \frac{T_\text {e}\ln \left( p_\text {ini}L_\text {d}\right) }{n_\text {li}}+1\right) \left( \frac{T_\text {g}\ln \left( p_\text {ini}L_\text {d}\right) }{n_\text {li}}+1\right) }{L_\text {i}}+1\right) \right. \\&\left. +n_\text {d}p_\text {ini}L_\text {d}\cdot \left( \dfrac{\left( \frac{T_\text {e}\ln \left( p_\text {ini}L_\text {d}\right) }{n_\text {li}}+1\right) \left( \frac{T_\text {g}\ln \left( p_\text {ini}L_\text {d}\right) }{n_\text {li}}+1\right) }{L_\text {i}}\right. \right. \\&\left. \left. +\dfrac{T_\text {e}\cdot \left( \frac{T_\text {g}\ln \left( p_\text {ini}L_\text {d}\right) }{n_\text {li}}+1\right) }{L_\text {i}n_\text {li}}+\dfrac{T_\text {g}\cdot \left( \frac{T_\text {e}\ln \left( p_\text {ini}L_\text {d}\right) }{n_\text {li}}+1\right) }{L_\text {i}n_\text {li}}\right) \right) \\&+C_\text {hi}n_\text {hi}p_\text {ini}\cdot \left( \dfrac{L_\text {d}\cdot \left( \frac{T_\text {e}\ln \left( p_\text {ini}L_\text {d}\right) }{n_\text {li}}+1\right) \left( \frac{T_\text {g}\ln \left( p_\text {ini}L_\text {d}\right) }{n_\text {li}}+1\right) }{L_\text {i}}+1\right) \\&+C_\text {hi}n_\text {hi}p_\text {ini}L_\text {di}\cdot \left( \dfrac{\left( \frac{T_\text {e}\ln \left( p_\text {ini}L_\text {d}\right) }{n_\text {li}}+1\right) \left( \frac{T_\text {g}\ln \left( p_\text {ini}L_\text {d}\right) }{n_\text {li}}+1\right) }{L_\text {i}}\right. \\&\left. +\dfrac{T_\text {e}\cdot \left( \frac{T_\text {g}\ln \left( p_\text {ini}L_\text {d}\right) }{n_\text {li}}+1\right) }{L_\text {i}n_\text {li}}+\dfrac{T_\text {g}\cdot \left( \frac{T_\text {e}\ln \left( p_\text {ini}L_\text {d}\right) }{n_\text {li}}+1\right) }{L_\text {i}n_\text {li}}\right) \\&+{\lambda }_6\cdot \left( \dfrac{\left( \frac{T_\text {e}\ln \left( p_\text {ini}L_\text {d}\right) }{n_\text {li}}+1\right) \left( \frac{T_\text {g}\ln \left( p_\text {ini}L_\text {d}\right) }{n_\text {li}}+1\right) }{L_\text {i}}+\dfrac{T_\text {e}\cdot \left( \frac{T_\text {g}\ln \left( p_\text {ini}L_\text {d}\right) }{n_\text {li}}+1\right) }{L_\text {i}n_\text {li}}\right. \\&\left. +\dfrac{T_\text {g}\cdot \left( \frac{T_\text {e}\ln \left( p_\text {ini}L_\text {d}\right) }{n_\text {li}}+1\right) }{L_\text {i}n_\text {li}}\right) \\&+{\mu }_2n_\text {d}p_\text {ri}+\dfrac{{\lambda }_1\cdot \left( 1-p_\text {ini}\right) }{T_\text {d}}+G_\text {i}\cdot \left( 281n_\text {d}-n_\text {d}n_\text {li}\right) +{\mu }_1n_\text {d}+\dfrac{C_\text {ti}n_\text {d}}{T_\text {d}}\\&+C_\text {mi}n_\text {d}+\dfrac{{\lambda }_3}{N_\text {mi}}=0 \end{aligned} \end{aligned}$$

In the above scenario, even if we assumed $\lambda _j=0, \forall j$ (As $\lambda _j$ had the decision variable $L_{di}$ as noted above), then also numerically the left-hand side of Eq. (28) would not be zero as there were no negative terms and all the parameters have non-zero positive values. Thus, this problem could not be solved using the KKT conditions. Several authors noted that often solution techniques become so complex in non-linear optimization that the problem can only be addressed using search techniques to reach a near optimal or a local optimal condition. We designed two such search techniques heuristics: one uses the mid-points of the boundary points and the other one maximum ratio (right hand side over left-hand side of each constraint) among the constraints. These are presented below.

Upon examining the objective function in Eq. (1), we found that the variable terms $p_{ri}$, and $L_{ri}$ would not have an impact and $n_{di}$ would have limited impact on the objective function

This was because, from Eq. (8), we found that the term $n_{di}L_{di}$ was a constant. Using the right-hand side of Eq. (8) onto Eq. (9), it can be further shown that the term $L_{ri}n_{ri}$ is also a constant. Additionally, it can be assumed that $n_{di}=n_{ri}$. This was for the governments to allow a measured approach to ensure that the return of the workers was not rushed for the social wellbeing of the workers as well as to ensure that this would not further worsen the infection rate. Assuming $L_{di}n_{di}=T_{depart}$ and $L_{ri}n_{ri}=T_{return}$, the modified objective function is

29 $$\begin{aligned} E_{ei-mod}&= \left[ \{(L_i-T_{depart})*n_{li}+T_{return}*(n_{li}+n_{di})+(T_{depart}-T_{return})*281\}*G_i\right] \nonumber \\&\quad +\left[ \left( \frac{T_{depart}*C_{ti}}{T_{di}}\right) +\left( \frac{T_{return}*C_{ti}}{T_{ri}}\right) + \left( T_{depart}*C_{mi}\right) \right] \nonumber \\&\quad +\left\{ p_{ini}*L_{di}*(1+R_i)*C_{hi}*n_{hi}\right\} \end{aligned}$$

**Fig. 2:**
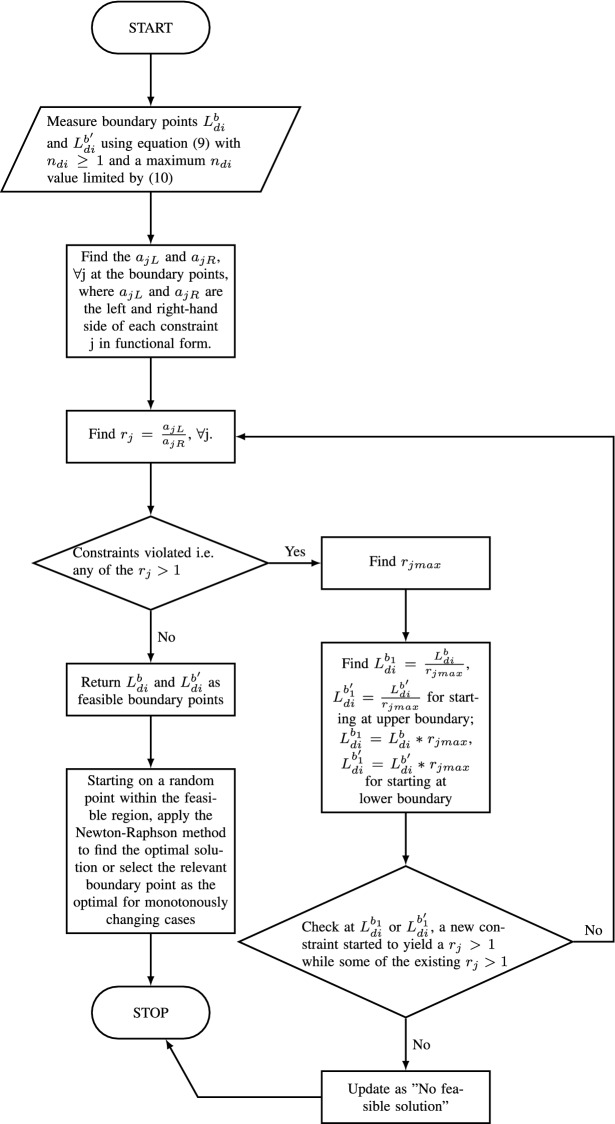
Search heuristics using the ratio of the right-hand side over the left-hand side of each constraint

We propose the following search techniques to solve the problem in Figs. 1 and 2.

In this particular minimization problem, the objective function can be shown to be monotonically decreasing in $L_{di}$ in the feasible region. Thus, we can find out the workable minimum value of the objective function using the upper boundary value of the feasible region. Consequently, the deduction of the lower boundary of the feasible solution could be ignored in this particular case.

#### Case II—migrant workers remaining in the workplace state

Unlike the last case, we considered that each state would ask their migrant workers to remain in the state they were working. The states would set up COVID-19 relief centers or ask the migrant workers to stay in their homes or a combination of both. The decision to split the proportion between the two options (migrant workers’ homes or relief shelters) was considered based on the following factors: its impact on overall economic expenditure, budget availability, the impact on the R rate, and the indirect impact on hospital admission due to the larger population being exposed to infection from living in confined places. Moreover, we also considered the maximum number of people, who can be given shelter in a particular area considering the UNHCR guidelines. More details are presented in the numerical analysis section. Similar to the migrant workers traveling interstate case, we assumed the key objective was to minimize the economic impact of this operation. This overall economic impact was taken from the expenditure to operate relief shelters (the first element is the number of people to be given shelter multiplied by the sum total cost of food and running the shelter), the cost of subsidizing the workers who would be asked to stay at home (the second element is derived by multiplying the number of people staying at home by the subsidy to be paid by the government per day and number of days of lockdown), cost to manage the hospital admissions (the third element is derived by multiplying the total population with the probability of being infected, then further multiplied by (1+$R_i$), the hospital cost per day and the total number of days to be treated at the hospital; the (1+$R_i$) captured the secondary infection from the infected population), and the economic loss due to the workers not working for the duration of the lockdown (the fourth element is derived by multiplying the total worker population in state i with the number of days of lockdown and GDP contribution per worker per day). The minimization of this economic impact was also constrained in this case. The left-hand side of constraint (31) represents the total number of people likely to be hospitalized which is constrained by the maximum number of hospital beds that can be arranged or are available. Applying a similar calculation from the case of migrant workers being transported to their state of domicile to this case, we get the constraint noted in (31). The left-hand side of constraint (32) denotes the number of people in each relief camp can be accommodated (by dividing the total number of people to be given shelter in relief camps by the capacity of each camp following UNHCR safety guidelines). This is constrained by the maximum number of people who can be given shelter in relief camps available in each state following the UNHCR guidelines. The first three blocks of elements of the objective functions are cost to the government of that particular state. The sum total of these costs is constrained by the total budget available for this. This was captured in constraint (33). The model is presented below.

30 $$\begin{aligned} \min : E_{si}&=\left[ \left\{ L_{ci}*\left( C_{ci}+C_{fi}\right) +\left( L_{hi}*C_{si}\right) \right\} *n_{li}\right] \nonumber \\&\quad +\left\{ p_{ini}*L_{ci}*(1+R_i)*C_{hi}*n_{hi}\right\} +(L_i*n_{li}*G_i) \end{aligned}$$

S.t.

31 $$\begin{aligned}&(p_{ini}*L_{ci})*(1+R_i) \le \frac{N_{hi}n_{li}}{(n_{li}+n_{hi})} \end{aligned}$$

32 $$\begin{aligned}&\frac{L_{ci}}{\rho _{ci}}\le N_{ci} \end{aligned}$$

33 $$\begin{aligned}&\left[ \left\{ L_{ci}*\left( C_{ci}+C_{fi}\right) +\left( L_{hi}*C_{si}\right) \right\} *n_{li}\right] +\left\{ p_{ini}*L_{ci}*(1+R_i)*C_{hi}*n_{hi}\right\} \le B_i\end{aligned}$$

34 $$\begin{aligned}&R_i=\left\{ (1+\lambda * T_e)*(1+\lambda *T_g)\right\} *x \le 1\end{aligned}$$

35 $$\begin{aligned}&L_{ci}+L_{hi} = p_{li}*L_i \end{aligned}$$

36 $$\begin{aligned}&L_{ci}, L_{hi} \ge 0 \end{aligned}$$

Similar to the earlier case of migrant workers travelling interstate, the R value considered in (34) was based on the calculations provided by Fang et al. (2020) following the SEIR model. The $\lambda$ is considered as $\frac{\ln (Y(t))}{t}$ (Fang et al., 2020). For this case II, we have two different alternatives: to allow the migrant workers to stay at their homes with further governmental support for their daily maintenance or offer them shelters in relief centers. Although in both cases there was a chance of the infection spreading, this risk would be significantly lower if the migrant workers were allowed to stay in their homes. Thus, this value could be considered as zero for our calculation as we are trying to propose a solution where the spread of the pandemic was in its early stage. Hence, we can consider $Y(t)= p_{ini}*L_{ci}$.

Case II migrant workers remaining in the state where they worked is a non-linear optimization problem. The objective function in Eq. (30), and the constraints (31), (33) and (34) are non-linear. Again, this followed the definition of the R value from Fang et al. (2020) which has a logarithmic term of the decision variable of the number of people likely to be infected as noted earlier. We tested the Karush Kuhn Tucker (KKT) conditions. To test the KKT conditions, we assume the following

37 $$\begin{aligned}&g_1=(p_{ini}*L_{ci})*(1+R_i) - \frac{N_{hi}n_{li}}{(n_{li}+n_{hi})}\end{aligned}$$

38 $$\begin{aligned}&g_2=\frac{L_{ci}}{\rho _{ci}}- N_{ci}\end{aligned}$$

39 $$\begin{aligned}&g_3=\left[ \left\{ L_{ci}*\left( C_{ci}+C_{fi}\right) +\left( L_{hi}*C_{si}\right) \right\} *n_{li}\right] +\left\{ p_{ini}*L_{ci}*(1+R_i)*C_{hi}*n_{hi}\right\} - B_i\end{aligned}$$

40 $$\begin{aligned}&g_4=\left\{ (1+\lambda * T_e)*(1+\lambda *T_g)\right\} *x - 1 \end{aligned}$$

41 $$\begin{aligned}&h_1= L_{ci}+L_{hi}- p_{li}*L_i \end{aligned}$$

Thus, the Lagrange multiplier function becomes

42 $$\begin{aligned}&\theta _{si}= E_{si}+\sum \lambda _j g_j+\sum \mu _k h_k \quad \forall \, \text {j, k} \end{aligned}$$

Considering the 2nd differential, the above Lagrange function is shown to be convex. Thus, if a solution exists, then we can discover a global solution by using the KKT conditions as seen below.

43 $$\begin{aligned}&\textit{Optimality conditions} \qquad \qquad \frac{\delta E_{si}}{\delta L_{ci}}+\sum \lambda _j \frac{\delta g_j}{\delta L_{ci}}+\sum \mu _k \frac{\delta h_k}{\delta L_{ci}}=0, \, \, \quad [\forall \, \text {j, k}] \end{aligned}$$

44 $$\begin{aligned}&\textit{Feasibility conditions} \qquad \qquad g_j+s_j=0 \, \, \text {and}\, \, h_k=0, \, \, \quad [\forall \, \text {j, k}] \end{aligned}$$

45 $$\begin{aligned}&\textit{Complementary slackness conditions} \qquad \qquad s_j\lambda _j=0, \, \, \quad [\forall \, \text {j}] \end{aligned}$$

Using the optimality condition, we obtain the following two equations

46 $$\begin{aligned}&\begin{aligned}&\left( \left( 4C_\text {hi}*T_\text {e}*T_\text {g}*n_\text {hi}+2T_\text {e}*T_\text {g}*{\lambda }_1\right) *p_\text {ini}*{\rho }_\text {ci}*L_\text {ci}+T_\text {e}*T_\text {g}*{\lambda }_4*{\rho }_\text {ci}\right) *\ln ^2\left( p_\text {ini}*L_\text {ci}\right) \\&\quad +\left[ \left( \left( \left( 4C_\text {hi}*T_\text {g}+4C_\text {hi}*T_\text {e}\right) *n_\text {hi} +\left( 2T_\text {g}+2T_\text {e}\right) {\lambda }_1\right) *p_\text {ini}*{\rho }_\text {ci}*t\right. \right. \\&\quad \left. +\left( 4C_\text {hi}*T_\text {e}*T_\text {g}*n_\text {hi}+2T_\text {e}*T_\text {g}*{\lambda }_1\right) *p_\text {ini}*{\rho }_\text {ci}\right) *L_\text {ci} \\&\quad \left. +\left( T_\text {g}+T_\text {e}\right) *{\lambda }_4{\rho }_\text {ci}t+2T_\text {e}*T_\text {g}*{\lambda }_4 *{\rho }_\text {ci}\right] *\ln \left( p_\text {ini}*L_\text {ci}\right) \\&\quad +\left( \left( 4C_\text {hi}*n_\text {hi}+2{\lambda }_1\right) *p_\text {ini}*{\rho }_\text {ci}*t^2 +\left( \left( 2C_\text {hi}*T_\text {g}+2C_\text {hi}*T_\text {e}\right) \right. \right. \\&\quad \left. \left. *n_\text {hi}+\left( T_\text {g} +T_\text {e}\right) *{\lambda }_1\right) *p_\text {ini}*{\rho }_\text {ci}*t\right) *L_\text {ci} \\&\quad +\left( \left( \left( 2C_\text {hi}*L_\text {i}*n_\text {hi}+L_\text {i}*{\lambda }_1\right) *p_\text {ini} +\left( \left( C_\text {fi}+C_\text {ci}\right) *L_\text {i}*{\lambda }_3+\left( C_\text {fi}+C_\text {ci}\right) *L_\text {i}\right) *n_\text {li}+{\lambda }_4\right) \right. \\&\quad \left. *{\rho }_\text {ci}+L_\text {i}*{\lambda }_2\right) *t^2\\&\quad +\left( T_\text {g}+T_\text {e}\right) *{\lambda }_4*{\rho }_\text {ci}*t =0 \end{aligned} \end{aligned}$$

47 $$\begin{aligned}&\left( C_\text {si}{\lambda }_3+C_\text {si}\right) n_\text {li}=0 \end{aligned}$$

In the current scenario, even if we assume $\lambda _j=0, \forall j$ (As $\lambda _j$ has the decision variable $L_{ci}$ as noted above), then also numerically the left-hand side of Eq. (46) would also not be zero as there are no negative terms and all the parameters have positive values. Equation (47) cannot be satisfied as the parameters are non-zero even if we assume $\lambda _3=0$. Thus, this problem could not be solved using the KKT conditions. We used the same search techniques as proposed in the case of the migrant workers traveling to their state of domicile in Sect. 3.3.1 with $L_{ci}$ instead of $L_{di}$ as the variable for which the search was conducted. Because the total number of people to be given shelter and asked to be staying at home should be equal to the total number of migrant workers who require assistance at a particular time, it is realistic to assume that the equality constraint in Eq. (35) is a binding one. For the purpose of mathematical simplicity, we replace $L_{hi}$ by making it a function of $L_{ci}$ (i.e. $L_{hi}=p_{li}L_i-L_{ci}$) from Eq. (35) to apply the search techniques noted in Sect. 3.3.1.

## Numerical analysis

In order to validate the models described in Sect. 3.3, we conducted numerical experiments. This section discussed these numerical experiments in Sects. 4.2, and 4.3. Furthermore, we conducted a sensitivity analysis to investigate how the models behave when some of the initial conditions are changed. These are presented in Sects. 4.4.1, 4.4.2. Finally, we compared the results derived from various numerical experiments to explore the implications for the decision-makers in Sect. 4.5. The numerical details of the model parameters are noted in Sect. 4.1.

### Assumed module parameter values

We derived the required parameter values from the conceptualization of the objective functions and relevant constraints. The details of these were explained in Sects. 3.3.1 and 3.3.2 respectively. One of the key features of this research is data-driven decision-making. The relevant data is collected from various sources such as governmental and non-governmental databases, data published in new media, and peer-reviewed journal articles among various other sources. Where applicable, we made realistic assumptions based on circumstances similar to this or whatever conforms to the regulations (Please see the additional considerations in Sect. 4.1.1).

The data on parameters collected from various secondary sources are summarized in Table 3. Most of the financial data collected from various sources are in the Indian rupee (). For the understanding of readers across the globe, these have been converted to USD ($). The international currency market conditions determine the conversion rates which fluctuate every moment. We converted these to USD by assuming a close approximation of the current time conversion rate (At the time of research was conducted) of 1 US$= 75.

**Table 3:** Considered parameter values

Parameter	States			Source
	Maharashtra	Karnataka	Delhi NCR	
$$G_{i}$$	$11.6814	$ 23.1060	$ 10.4181	PRS India, (2019a, b, c)
$$L_i$$	1,800,000	900,000	600,000	Please see in Sect. 4.1.1
$$N_{ti}$$	50	50	50	Please see in Sect. 4.1.1
$$N_{ci}$$	2000	2000	2000	Please see in Sect. 4.1.1
$$N_{mi}$$	471	727	780	Please see in Sect. 4.1.1
$$N_{hi}$$	25,000	15,000	10,000	Please see in Sect. 4.1.1
$$M_i$$	86	53	26	Ministry of Health, India (2020)
$$\rho _{ci}$$	500	500	549	Please see in Sect. 4.1.1
$$B_i$$	$214.8 mil	$ 87.73 mil	$ 6.67 mil	Special-Correspondent (2020c)
$$C_{mi}$$	$33.33	$26.67	$32	Barnagarwala (2020), Indian Express (2020a), The Economic Times (2020)
$$C_{ti}$$	$11750.4	$11750.4	$11750.4	Dutta (2020)
$$C_{si}$$	$4.53	$6	$7.33	Jain (2019)
$$C_{fi}$$	$1.87	$1.6	$1.87	Times of India (2020)
$$C_{ci}$$	$2.43	$1.95	$2.46	Please see in Sect. 4.1.1
$$C_{hi}$$	$120	$114.33	$133.33	Mumbai Mirror (2020), Yasmeen (2020b), Saxena (2020)
$$T_{di}$$	1296	1296	1200	Indian Express (2020b)
$$T_{ri}$$	1728	1728	1728	Indian Express (2020b)
$$p_{ini}$$	62.6/mil. population	4.34/mil. population	115.7/mil. population	Maji et al. (2020)
$$p_{ri}$$	0.45	0.45	0.45	Press Trust of India (2020c)
$$ p_{li}$$	0.66	0.67	0.65	Please see in Sect. 4.1.1

#### Additional considerations for parameters

1. There have been debates over the number of migrant workers who were staying in the selected states and how many of them were displaced due to lockdown among various sources available. Thus, we considered the overall numbers of migrant workers staying in each state ($$L_i$$) as realistic approximations based on information gathered from various sources (Chishti, 2020; Laskar, 2020; Kumar & Moudgal, 2020; Shrangi, 2020). These sources generally agreed that around 60–70% of migrant workers were displaced post-lockdown measures implementation. Again, we calculated the realistic approximation of the number of people who traveled interstate with government help ($$p_{li}$$) from the information gathered from these sources. We assumed a similar set of values for the proportion of migrant workers requiring government help to settle down if they were asked to stay in the state they were working.
2. The number of special trains Indian Railway operated from various states varied (Press Trust of India, 2020a; Special Correspondent, 2020a, f; Rohatgi, 2020). For a practical estimation, we assumed that each of these states can run up to 50 trains per day based on guideline numbers approved in another state (Press Trust of India, 2020b).
3. The nationwide lockdown started on 25th March, 2020 in India. The unlocking process took place over time and in some cases, regional governments needed to make decisions to avoid local outbreaks. However, from early June, 2020, the stepwise unlocking began in many areas of the country. Therefore, we approximated the number by assuming $$n_{li}$$= 60 days for our basic case.
4. The number of days for which the transportation operation of migrant workers was conducted, varied between different states. The Indian railways operated special trains for this operation called “Shramik Special”. It began around early May, 2020 and by the end of May 2020, a significant number of migrant workers had been transported (Nag, 2020). In some cases, there was a lack of a substantial number of passengers to travel Singh (2020a). The $$n_{di}$$ was derived from the model, and it was restricted by a certain proportion (y) of lockdown days. Considering the empirical observation in this case, we assumed this as 50% of the total number of lockdown days, namely 30 days. For practical purposes, it can be assumed that $$n_{di}=n_{ri}$$ as noted earlier.
5. According to an article published in the Economic Times (Mukherjea, 2019), the migrant workforce brings in approximately $$2\%$$ of the country’s overall economic contribution. Thus, we calculated the GDP or GSDP per worker capita by multiplying the state’s GSDP value available for the year 2019–20 by 2% and then dividing that number by the total number of migrant workers residing in that state. We further converted that number per day by dividing it by the number of days in a year.
6. According to a report published in the Hindustan Times (Dutta, 2020), it cost about $45.33 per person to travel on each train. In Table 3, we already mentioned the capacity of each train to be 1728. Thus, we multiplied this by $45.33 to get the total cost to operate the single train at full capacity. We assumed that it was the cost of running each train even if the train was operating at less than full capacity. Eighty-five percent of this cost was paid by the Railways leaving the rest of the 15% to be borne by each state. This is how we obtained the $11750.4 per train as the number.
7. The data on the capacity of testing that can be done by each ICMR approved testing center was difficult to be collected for various reasons since it changed over time with the increase in the number of people affected, the type of center, and resource availability among many others. Thus, we gathered data on the following: the number of tests conducted in the selected states (Sabrwal, 2020; Ghosh & Kapoor, 2020; Menon, 2020; Special Correspondent, 2020d) and the total number of ICMR-approved testing centers available (Including the private ones) in each state under consideration (Ministry of Health, India, 2020). Then we calculated the approximate capacity of each testing center ($$N_{mi}$$) by dividing the number of tests per day by the total number of centers.
8. Similar to the number of COVID-19 tests per day, the number of available dedicated COVID-19 hospital beds also emerged over time. In the beginning, the selected states had a lower number of beds available such as only 2490 in Karnataka (Government of Karnataka, 2020b). However, over time, with growth in the number of affected people, each state needed to increase the number (Debroy & Jain, 2020; Yasmeen, 2020a). We considered all these and also collected data from the local state government’s COVID Dashboard equivalent (Government of Maharashtra, 2020; Government of Karnataka, 2020a; Government of Delhi NCR, 2020). Subsequently, we summarized the numbers in Table 3, based on realistic assumptions up until the end of June 2020 from the information.
9. According to a report published in the Times of India (2020), the government of Maharashtra announced a subsidized food scheme called "Shiv Bhojan" which allows people to buy food at a subsidized rate of $ 0.13 per plate, costing the Government, appx. $ 0.67. Two meals per day, it should cost $ 1.34. However, we added some costs to cover additional subsistence expenditure to make the figure $ 1.87. Considering the similarities in most of the lifestyle expenditures, we assumed the same cost per person for food and other subsistence in Delhi NCR as $ 1.87. However, considering slightly less expensive food and other subsistence in Karnataka, we considered the total cost of subsistence in Karnataka as $ 1.6.
10. Each of the state governments considered in this research set up temporary relief shelters immediately after the pandemic started, such as 262 in Maharashtra for 70,000 stranded migrant workers (IANS, 2020), 193 in Karnataka (Special Correspondent, 2020e), and 224 in Delhi (The Times of India, 2020). For our proposed model in the case of remaining migrant workers, we assume that local state government COVID-19 teams would set up relief shelters in schools, and government buildings temporarily vacated due to the COVID-19 outbreak. According to Chattopadhyay (2020), in a report published in Indian Express, governments can acquire places to set up emergency measures to manage the pandemic as per the Epidemic Act, 1857. We assumed that in Maharashtra and Karnataka, up to 500 such relief shelters ($$\rho _{ci}$$) could be set up whereas we kept the same total number of 549 for Delhi NCR as highlighted in The Times of India (2020).
11. Various schools and various government premises may have different physical areas. Furthermore, it was difficult to collect such data accurately. However, there are some guidelines on minimum school areas required (Kakumanu, 2018; Mishra, 2020). Regarding these guidelines, we considered the relief camps would be 8000 sqm. and could serve a maximum of 2000 people ($$N_{ci}$$) i.e 1 person per 4 sqm area following the guidelines of UNHCR, The UN refugee agency (UNHCR, 2015).
12. We assumed $$T_e$$ as 5 days as the average latency period by which the symptoms would become visible. This was based on the commonly observable empirical cases worldwide. The average generation period for SARS was reported as 8.4 days in the study of Lipsitch et al. (2003). However, the authors also reported that the value was 10 days on average in the first two weeks. Since in this study, we are proposing the strategy for preventing the outbreak right from the onset, we considered $$T_g$$ as 10 days. For calculating the $$\lambda $$ value as mentioned in Sect. 3.3.2, we measured Y(t) based on the proportion of people infected as highlighted in Maji et al. (2020). The authors emphasized the numbers available at the end of 26th April 2020 which was approximately 1 month since the lockdown had started. We assumed the same values for our basic calculation even for a 60 days lockdown period. However, we presented additional cases in the sensitivity analysis to obtain different scenarios.
13. The non-food related expenditure ($$C_{ci}$$) in relief shelters would involve the cost of electricity and water.
  We calculated the per capita electricity consumption per day as 3.61 kWh, 3.03 kWh, and 4.2 kWh for Maharashtra, Karnataka, and Delhi NCR respectively from the guideline statistics available in sources Jaganmohan (2020a, b), Swamy et al. (2016) & Anon. (n.d.). We gathered the unit cost of electricity from Jain (2020b). Then we multiplied the unit charges to the per capita consumption per day to get an estimate for the electricity cost per person per day.
  We obtained the approximate per capita water consumption per day from Shaban and Sharma (2007) and the cost of water per litre from FE Online (2017), Deshpande (2019) & Express News Service (2020). Then we multiplied the per capita consumption per day by the unit rates to get the cost of water per person per day.

### Scenario 1: transportation of migrant workers

We used the two proposed search techniques to identify the optimal solution to the problems for the three selected states. To apply either or both techniques, boundary points of the feasible solution region were required. For the state of Maharashtra, the boundary point coordinates were derived as between 39,500 and 1,185,000, using Eq. (9) considering a minimum value of $n_{di}=1$ and a maximum value limited by constraint (10). Numerically it can be shown that the first order derivative of the objective function is negative in this interval with respect to $L_{di}$. Hence, the feasible maximum value in the interval that satisfied all the constraints should be the workable optimal solution, and any further application of techniques such as Newton–Raphson were not needed. Similar patterns were observed in the cases of the states of Karnataka, and Delhi NCR. The boundary points were found to be 606,667 and 20222 for the state of Karnataka and 390000 and 13000 for the state of Delhi NCR. We then applied the two search techniques described earlier.

**Fig. 3:**
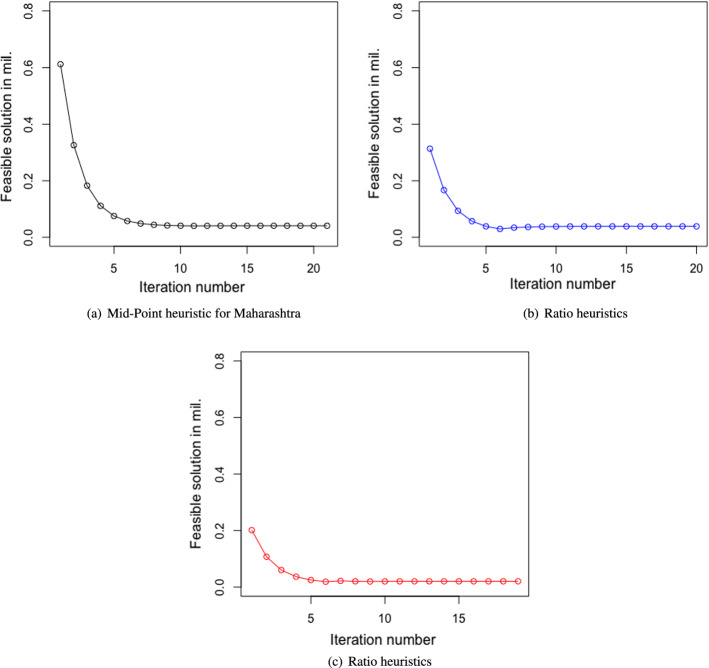
Results of mid-point heuristics

Both the heuristics offered the same solution. However, the heuristics using the maximum of the ratios required a fewer number of iterations in comparison to the heuristics using the mid-points of the boundary points to reach the exit conditions. This is good for the computational effort. In this particular case, for all three states, the heuristics based on the ratios, offered the results in one or two iterations, whereas the other method took a few iterations to reach the solutions. Some of the results of the iterations using the heuristics based on the mid-points are noted in Fig. 3a–c. However, the cases where the objective function is not monotonous, and both the (upper and lower) boundaries are required to be found, may require heavy computational efforts. In such cases, the feasible boundary points which are farthest from the initial boundary points may take a larger number of iterations than the mid-point heuristics. Consequently, in those cases, the computational efforts for the ratio-based heuristic will be higher in comparison to the heuristic based on the mid-point. The optimization problem we have can be solved using the designed heuristics. However, for a considerably more complex problem where neither the KKT condition exists nor it is easy to find the binding constraints, it would be difficult to design heuristics that are computationally efficient. In those cases, it would be easier to use evolutionary algorithms which use evolution in simulated environments to search for solutions to complex problems (Whitley, 2001). This search is based on Darwin’s survival of the fittest principle, allowing the opportunity to emerge for global optimal solutions for complex problems (Dasgupta & Michalewicz, 1997). Due to its capacity to solve complex optimization problems with the ability to offer globally optimal or near globally optimal solutions, these have been considered in various humanitarian logistics related research works such as Burkart et al. (2017). Among the evolutionary algorithms, the genetic algorithms have been one of the most commonly used for example for pre- and post-disaster related decision making (Agarwal et al., 2022). Thus, for this problem, we further conducted the analysis for a basic case using the genetic algorithm available in MS Excel with a mutation rate of 0.075, a convergence of 0.0001, and a population size of 100.

**Fig. 4:**
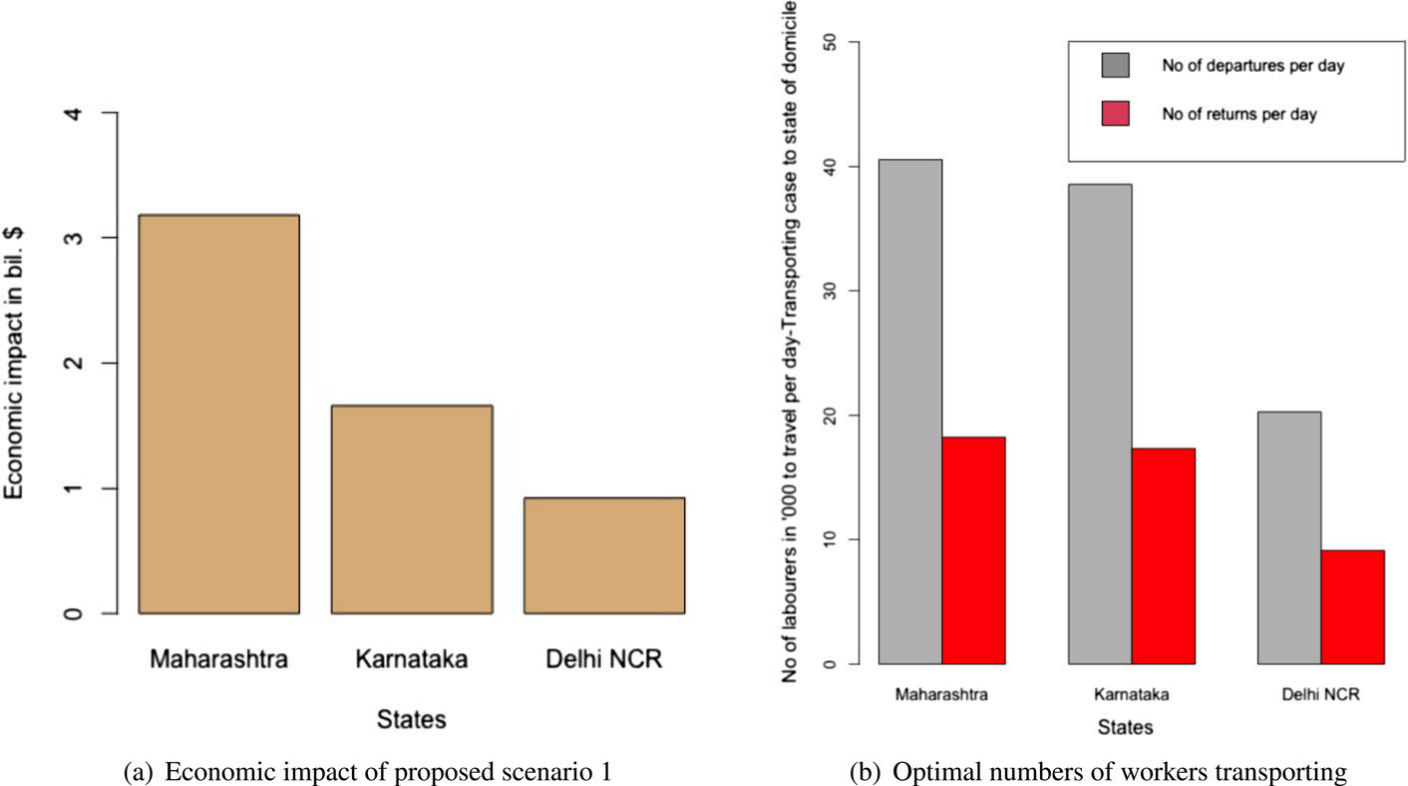
Results from proposed scenario 1: transportation of migrant workers interstate due to COVID Crisis

Table 4 and Fig. 4a and b present the following: the result for the total economic impact ($E_{ei}$), and the optimal values of the number of migrant workers can be sent back to their home state per day from state i ($L_{di}$), the number of migrant workers per day can be brought back to state i ($L_{ri}$), the number of days the migration movement to be run for the departure from state i ($n_{di}$), and the number of days the migration movement to be run for the return to state i ($n_{ri}$). The numerical values of the left-hand side of the constraints mentioned in Eqs. (2)–(7) are presented in Table 5.

Comparing the results from Table 5 with the values presented in Table 3, we found that all the constraints are satisfied except for the budget constraints for Delhi NCR as seen in Table 5. This means the current budget allocation by the Government of Delhi NCR would not be sufficient to carry out this strategy and the government required a greater budget.

**Table 4:** Results of the optimal values of the decision variables and the objective function of scenario 1

	Maharashtra	Karnataka	Delhi NCR
$$E_{i}$$	$3.18 bil	$ 1.66 bil	$ 0.92 bil
$$L_{di}$$	40485	38531	20272
$$L_{ri}$$	18218	17340	9123
$$n_{di}$$	30	16	20
$$n_{ri}$$	30	16	20

**Table 5:** Results of the constraints for scenario 1

LHS of the constraint	Maharashtra	Karnataka	Delhi NCR
Eq. (2)	31	22	17
Eq. (3)	11	11	6
Eq. (4)	86	53	17
Eq. (5)	81	3	49
Eq. (6)	0.097	0.05	0.072
Eq. (7)	$ 0.054 bil	$ 0.022 bil	$ **0.017 bil.**

The bold letters in the table are to demonstrate that the corresponding budget constraint has been violated

### Scenario 2: migrant workers remaining in their workplace state

**Fig. 5:**
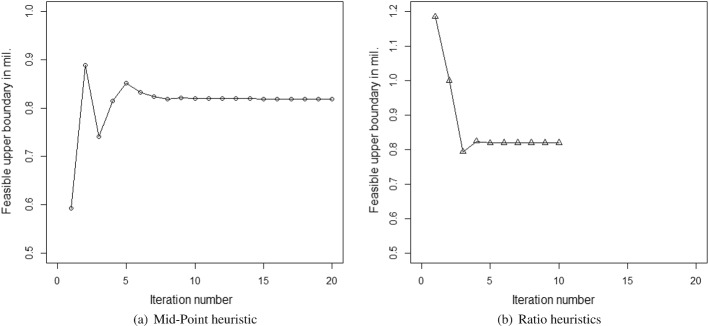
Results of heuristics for Maharashtra

**Fig. 6:**
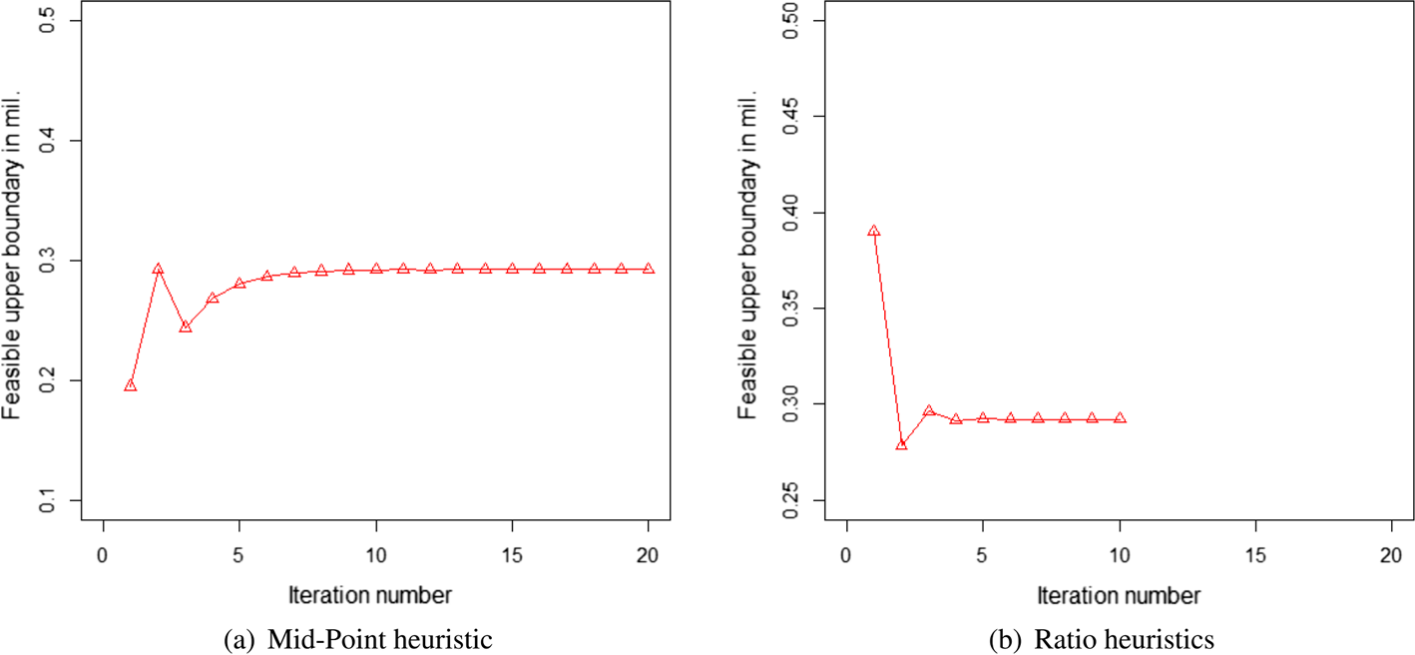
Results of heuristics Delhi

We applied both the earlier mentioned search instructions/heuristics to the three cases for Maharashtra, Karnataka, and Delhi NCR respectively. The results of the iterations are shown in Figs. 5a, b, 6a, and b.

For Maharashtra, the boundary point coordinates for $L_{ci}$ are 0 and 1185000 respectively. At $L_{ci}=0$, any real solution is not feasible due to the involvement of the logarithmic function in the objective function. Thus, we reset the boundary as 1 as that is the next possible number $L_{ci}$ can assume. Applying the above heuristics,

we found the upper bound of the feasible solution of $L_{ci}$ would be 819338. Numerically, it can be shown that the first-order derivative (with respect to $L_{ci}$) is negative throughout this feasible region and thus, it would be a monotonously decreasing function of $L_{ci}$. Hence, without the need for applying the Newton-Raphson method, we can use the feasible upper boundary as the workable optimal solution in this case, namely $L_{ci}=819338$ is the workable optimal solution. From Eq. (35), we derive optimal $L_{hi}=365662$. If we had a scenario where the objective function was not monotonously decreasing in the feasible region, then the Newton-Raphson or other search techniques would have been required to obtain the workable optimal solution.

Regarding Karnataka’s case, we start with identifying the boundary points of $L_{ci}$ from the constraint Eq. (35) as 0 and 606667. It can be seen again that the first-order derivative for the objective function is negative with respect to $L_{ci}$. More interestingly, due to the very low probability of infection, none of the constraints is violated at the upper boundary $L_{ci}=606667$. Thus, it can be considered as the workable optimal solution without any further calculation.

For Delhi NCR’s case, the boundary points from Eq. (35), are 1 (as $L_{ci}=0$ leads to infeasible values) and 390000 for $L_{ci}$. On both the boundaries, the constraint $g_3$ (Budget) has been violated and at $L_{ci}=390000$, the constraint $g_4$ (R number) is also violated. This would mean that it is not possible to find a feasible region with all the current set of constraints satisfied because the budget constraint would be violated for $1 \le L_{ci} \le 390000$. Assuming, the government would be interested to curb the spread of the disease, we allowed the budget constraint to be relaxed for this case. Thus, if we drop the constraint $g_3$ from the list of constraints, then $L_{ci}=1$ is the boundary where none of the constraints is violated. Thus, applying the designed instructions, we obtain the results of the iteration as noted in diagrams 6(a) and b.

Regarding Delhi, we found that constraints are satisfied between 0 and 292303. It can be further shown that the objective function is monotonously decreasing in this feasible region. Thus, the optimal solution would be at $L_{ci}=292303$. Moreover, the amount of violations for the budget constraint is also minimum at this point. Thus, from Eq. (35), we derive the workable optimal $L_{hi}=97697$.

**Fig. 7:**
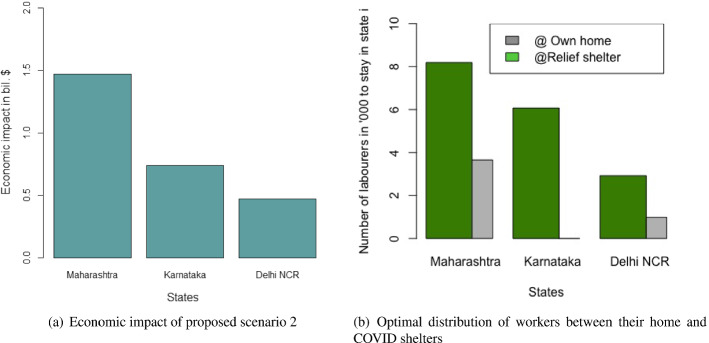
Results for the proposed scenario 2: migrant workers remaining in the state where they work

Both the heuristics offered the same solution. Similar to the case of migrant workers traveling, the heuristics using the maximum of the ratios required a lesser number of iterations in comparison to the heuristics using the mid-points of the boundary points to reach the exit conditions. Again, this is good for the computational effort. However, as discussed earlier, the cases where the objective function is not monotonous, and both the (upper and lower) boundaries are required, may require heavy computational efforts. In such cases, this advantage of less computational effort of the ratio-based approach may not be valid. Again, a significantly complex problem could be solved more easily using the evolutionary algorithms as mentioned in the case of the migrant workers who traveled. The results are presented in Table 6, and Fig. 7a and b. The remaining results are presented in Table 7 which confirms the validity of the results we derived from the heuristics.

**Table 6:** Results of the optimal values of the decision variables and the objective function of scenario 2

	Maharashtra	Karnataka	Delhi NCR
$$E_{i}$$	$ 1.47 bil	$ 0.74 bil	$ 0.47 bil
$$L_{ci}$$	819338	606667	292303
$$L_{hi}$$	365662	0	97697

The economic impact would have been less by keeping the migrant workers in the COVID-19 relief shelters provided by the local governments of each state to the maximum of 2000 people in each shelter. However, that was found to impact the R-value for the cases of Maharashtra and Delhi NCR. As a result, the optimal solutions were divided between keeping the migrant workers in relief camps and as well as in their own homes supported by governmental aids. On the contrary, the situation was different in the case of Karnataka, because the proportion of infection in Karnataka in the earlier stage of the pandemic in India was very low. As a result, the impact on the R-value was significantly low and the optimal solution was fully in favor of keeping the migrant workers in relief shelters because it was economically less expensive.

**Table 7:** Results of the constraints for scenario 2

LHS	Maharashtra	Karnataka	Delhi NCR
Eq. (31)	102	4	68
Eq. (32)	1639	1214	532
Eq. (33)	$ 0.211 bil	$ 0.071 bil	$ **0.087 bil.**
Eq. (34)	1.00	0.003	1.00

The bold letters in the table are to demonstrate that the corresponding budget constraint has been violated

### Sensitivity analysis

#### For the transportation of migrant workers case

**Fig. 8:**
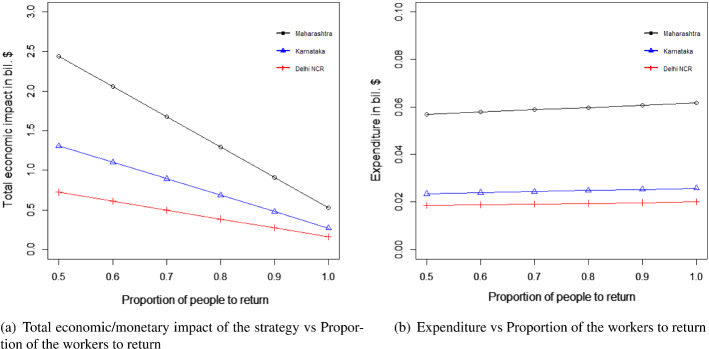
Results for the sensitivity of total cost by changing the proportion of people to return post lockdown

We changed the proportion of migrant workers who wanted to return post lockdown. The results for the total expenditure and the overall economic impact are presented in Fig. 8a and b. With the increase in the proportion of migrant workers who wished to return, the total economic cost decreased. This was because, with the increase in the number of migrant workers returning to the state, the impact on the total economic loss was reduced. This improvement was found to be more than the increasing expense of transportation to bring the migrant workers back to state i. This can be explained by Eq. (1).

**Fig. 9:**
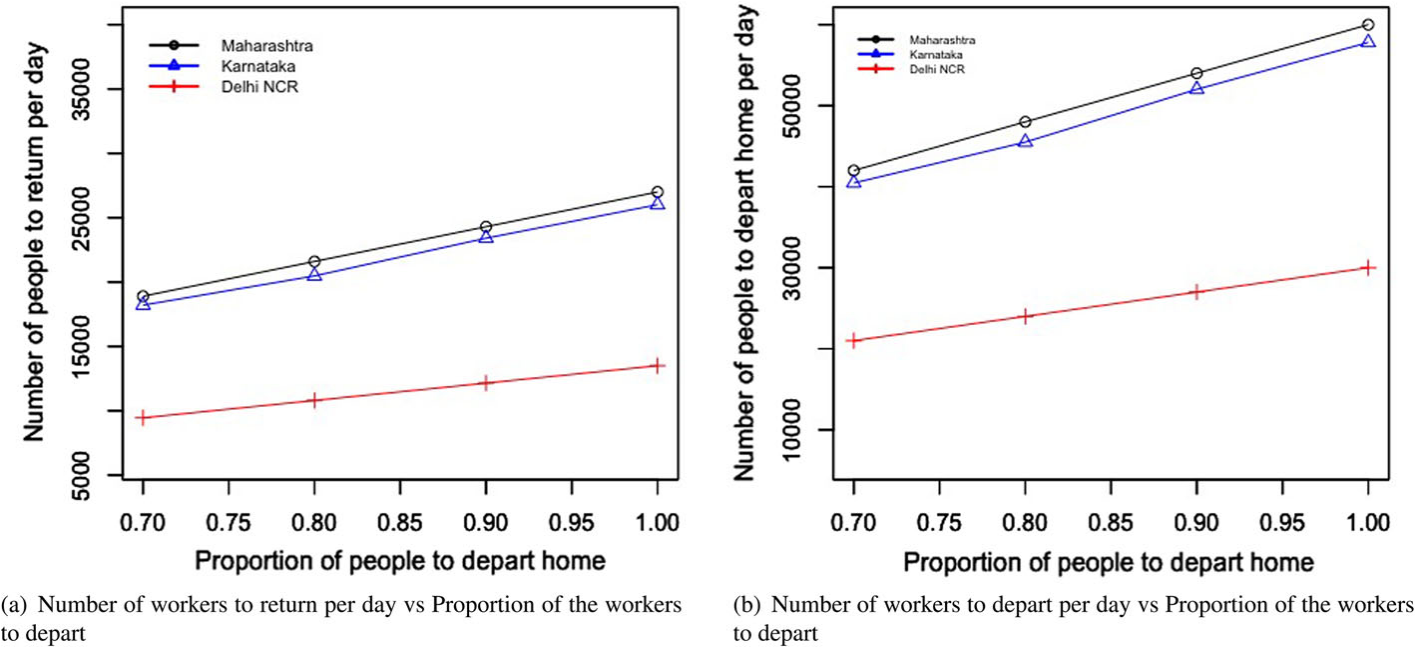
Results for the sensitivity of the decision variables by changing the proportion of migrant workers to depart due to lockdown

**Fig. 10:**
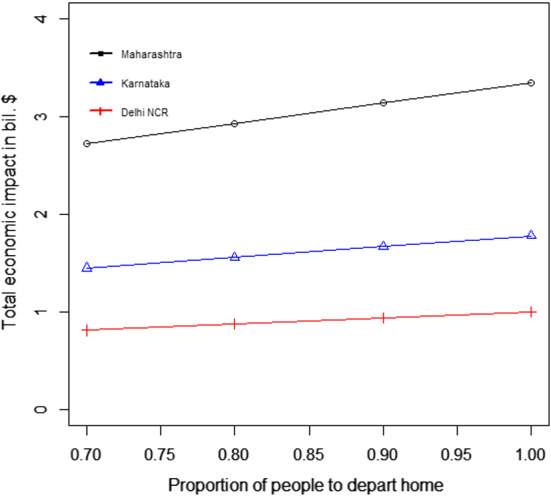
Sensitivity of the overall economic impact for changes in the proportion of migrant workers travelling

**Fig. 11:**
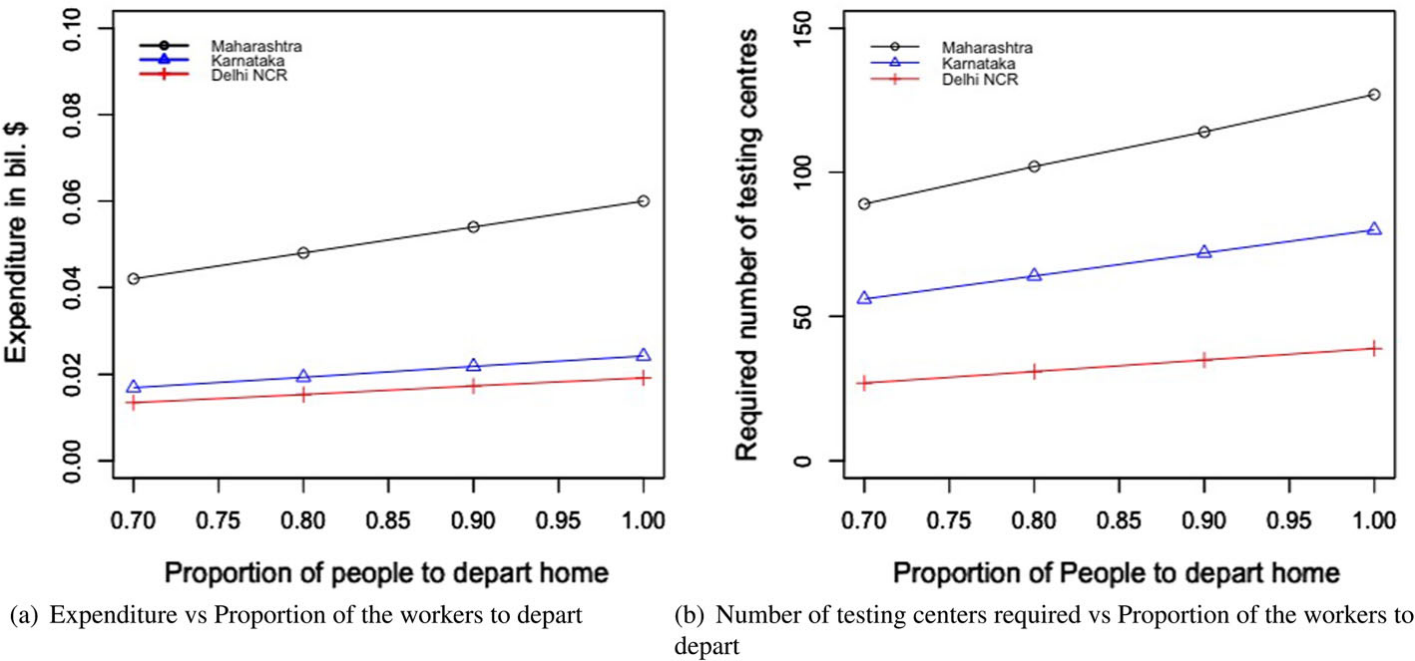
Results for the sensitivity of total expenditure and the number of testing centers required by changing the proportion of people to depart due to lockdown

**Fig. 12:**
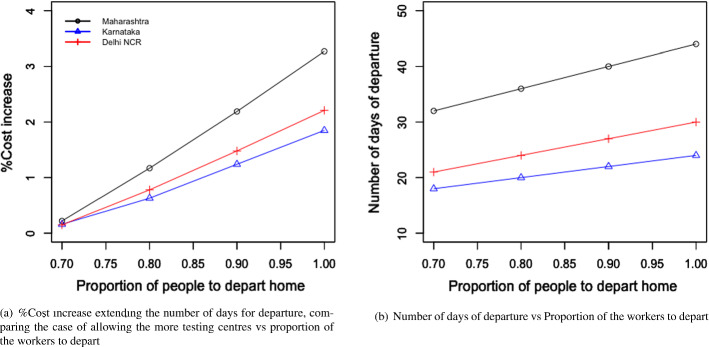
Results for the sensitivity of the number of days the government can allow the departure transportation operation to continue, and subsequent cost increase by changing the proportion of migrant workers to depart due to lockdown

**Fig. 13:**
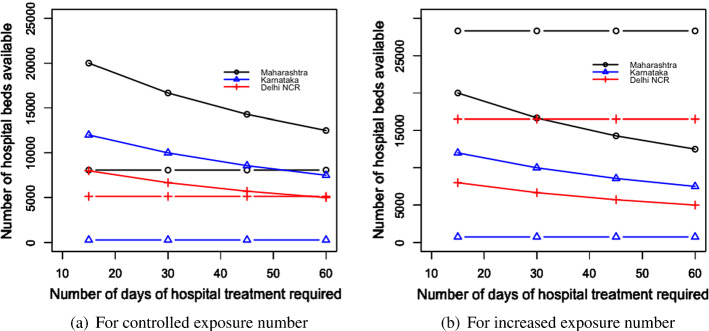
Results for the sensitivity of the number of hospital beds required by changing the number of days of hospitalisation (The solid lines denote the available number, where as the dashed lines represent the required number)

The results for the changes in the number of workers to return per day, the number of workers to depart per day, and the changes in the total economic impact are respectively presented in Figs. 9a, b, and 10. However, with the increase in the percentage of the worker population who expressed an interest in traveling, some of the constraints were violated. For the state of Maharashtra, the number of testing centers at the current capacity was found to be insufficient to conduct the required number of test if there were an increase in the number of workers migrating per day (Please see Fig. 11b). Moreover, the budget allocated for Delhi NCR was already less than the required amount to carry out the base case. This would be even more restrictive with the increase in the percentage of migrating workers (Please see Fig. 11a). The option for the government was to either allow the number of days for the operation to continue to be raised ($n_{di}$) keeping the number of traveling workers ($L_{di}$) the same as the base value $n_{di}$ or setting up additional facilities to test more workers. The results for changes in $n_{di}$ are shown in Fig. 12b. Further analysis revealed that the option to set up additional medical testing centers would marginally cost less than stretching the number of days ($n_{di}$) to allow the migrant workers to travel shown in Fig. 12a. We conducted a sensitivity analysis on the number of days of hospital treatment required. In the base case, the R value of the infection was low. The number of hospital beds required was mostly lower than the number of hospital beds available even though there was a gradual reduction in availability with the increase in the number of hospitalization days. This was even mostly true for the cases where the probability of infection was increased. The only exception was the case of Delhi NCR where the probability was increased by 100 times the base case and the number of days of hospital treatment required was 60 days. Thus, we are only showing the results for $p_{ini}$ increased by 100 times for various numbers of days of hospitalization. The results are presented in Fig. 13a. Further investigation was conducted by removing the assumption that only those workers who are due to travel would leave their homes to travel following government advice. Further analysis revealed that the situation could become completely out of control if the number of workers exposed to the disease increased because of the overcrowding of those hoping to travel. Hospital availability was consistently lower than the required for both Maharashtra and Delhi NCR if the probability of infection increased the base case by 100 times. The results are presented in Fig. 13b. This reemphasizes the importance of strict control to be implemented by the government to ensure only those workers scheduled to travel on a particular day should leave their homes.

**Fig. 14:**
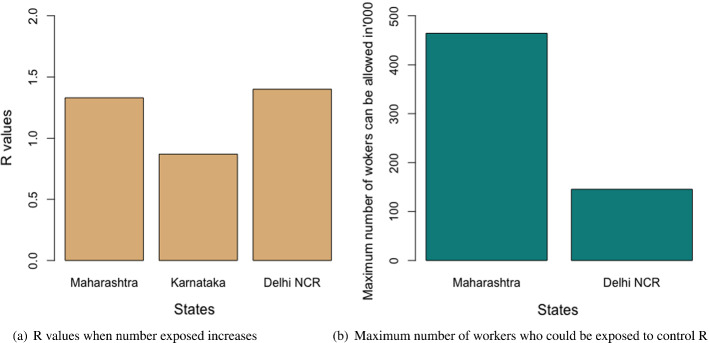
Results for the sensitivity of the R value of the infection

Further analysis was conducted on the R value particularly on the number of workers susceptible to the disease due to exposure. Earlier, we considered that the government in each state would only allow $L_{di}$ number of people due to travel that day to leave their homes, to control the exposure to COVID-19 through less congestion. However, this was paradoxical under the humanitarian situation of such considerable uncertainty, due to various reasons such as socio-cultural, demographic, and infrastructural challenges (e.g. a significantly high number of migrant workers, and challenges of information communication channels due to lack of internet access among workers) among many other relevant issues. As a result, it was very highly likely, that there would be more people than $L_{di}$ who would turn up with the hope of traveling. This could lead to a higher number of people exposed to the disease and their susceptibility. If all the migrant workers who required government assistance to travel inter-state became exposed to the disease, then particularly regarding Maharashtra and Delhi NCR, the R-value of the infection would be much more than 1 (Please see Fig. 14a). In the case of the state of Karnataka, the R value was marginally less than 1. However, at the early stage of infection spreading rapidly, this situational difference in R value could soon disappear leading to a faster and greater spread of the infection. We calculated the number of workers who could be allowed before the R value became more than 1 presented in the Fig. 14b.

#### For the migrant workers staying in the work state’s case

**Fig. 15:**
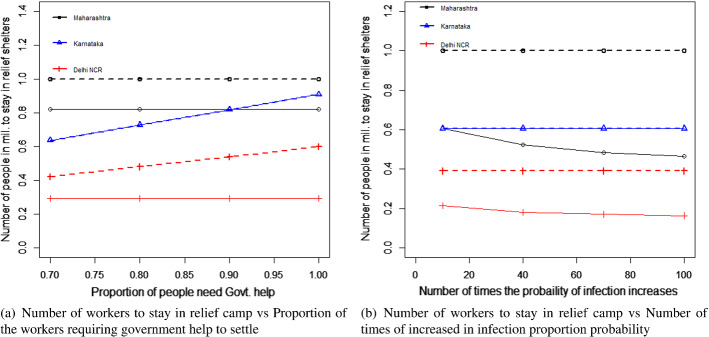
Sensitivity for number of the workers to stay in relief camps

**Fig. 16:**
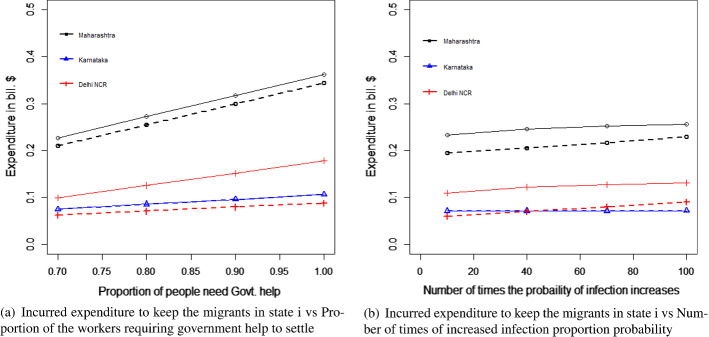
Sensitivity for the expenditure incurred to keep the migrant workers in state i

**Fig. 17:**
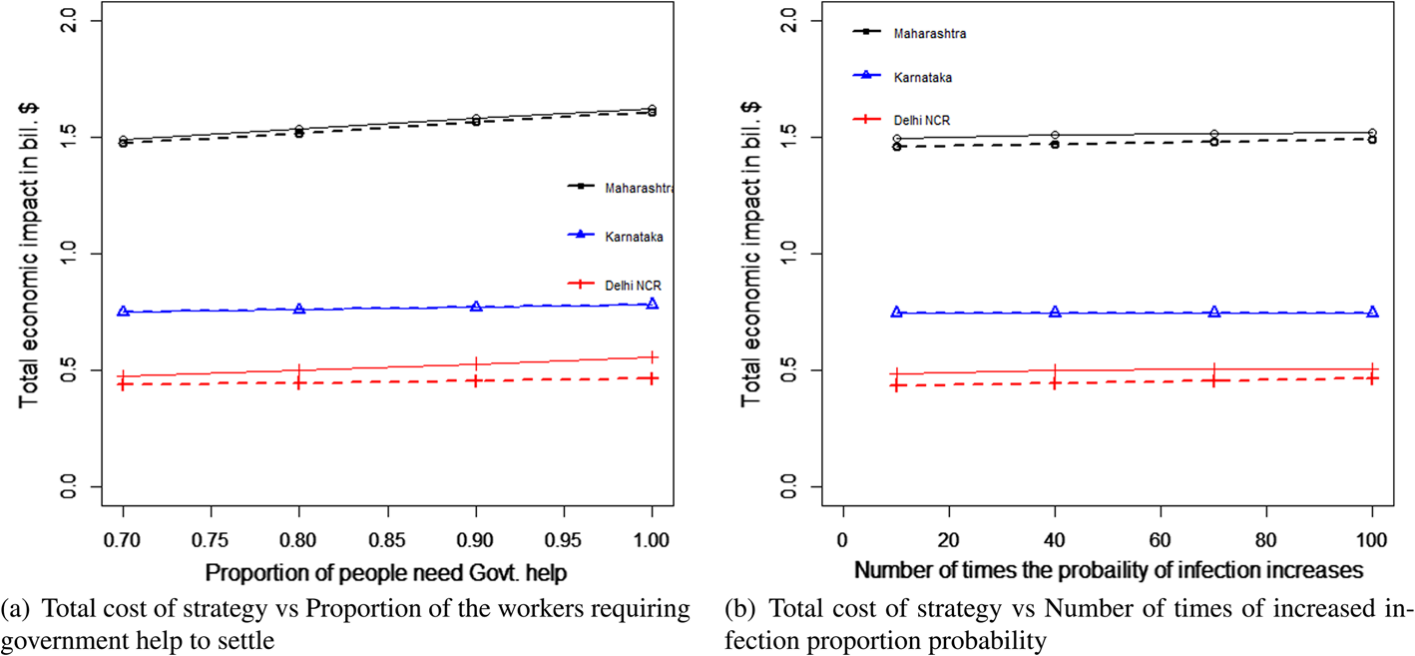
Sensitivity for the total economic cost of the strategy to keep the migrant workers in state i

**Fig. 18:**
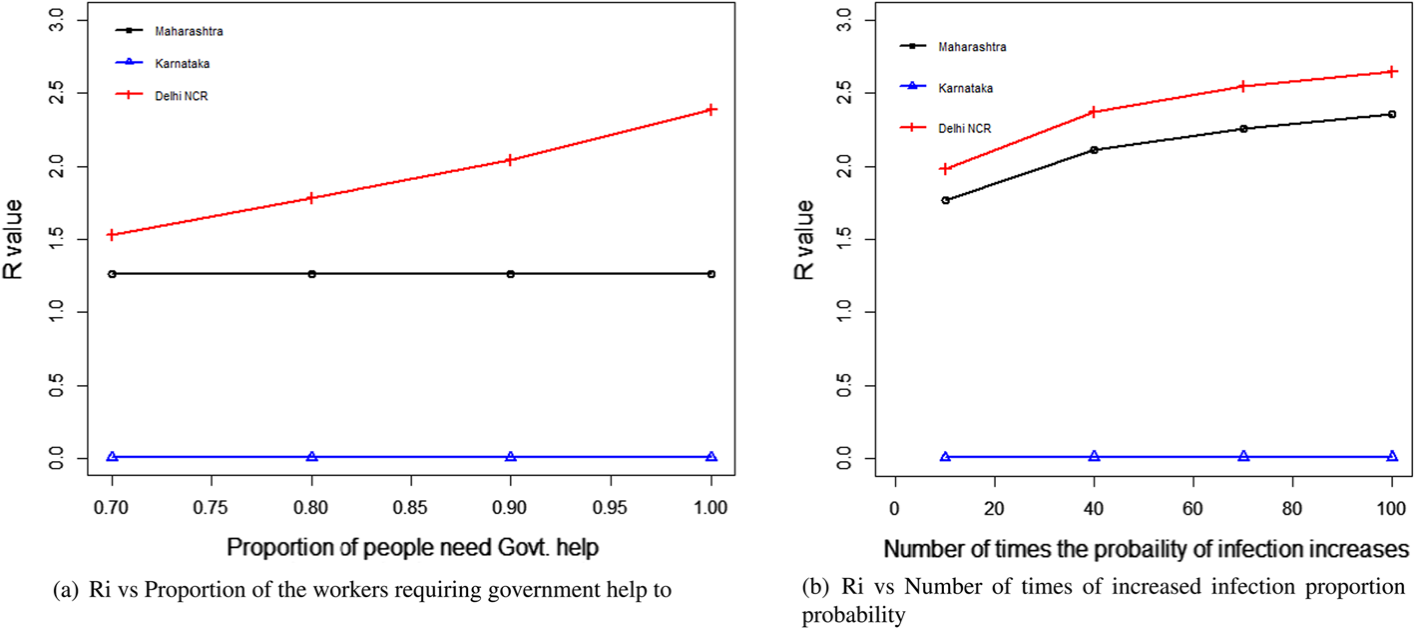
Sensitivity for the R values, to keep the migrant workers in state i

We extended the analysis presented in Sect. 4.3 in two important areas: by changing the proportion of workers requiring government help ($p_{li}$) and by changing the probability/proportion of infection/infected population ($p_{ini}$) as suggested by Maji et al. (2020). The results are presented in Figs. 15a, b, 16a, b, 17a, b, 18a, and b. In many cases, the budget and/or the R number constraints were found to be violated with the increase in the proportion of workers requiring government help to settle down due to the pandemic of COVID-19. We conducted the sensitivity by relaxing either or both constraints depending on the case. The solid lines in Figs. 15a, b, 16a, b, 17a, and b represent the cases where the budget constraints were relaxed to control the R value of the infection. Conversely, the dashed lines were used to represent the relaxation of the R number (except in the case of Delhi NCR where both had to be relaxed due to constraint violation). We only plotted the changes in R values for the case when the constraints of R values were allowed to be relaxed for two sensitivity issues addressed here [(changing the proportion of workers requiring government help ($p_{li}$) and changing the probability/proportion of infection/infected population ($p_{ini}$)] in Fig. 18a, and b. This was mainly because of allowing the budget constraint to change; R value could be controlled within 1. When the constraints for R were relaxed, with the increase in the proportion of workers requiring government intervention to settle down, the optimal solution was in favor of keeping more people in relief shelters. The number of workers in each relief shelter could be increased up to a maximum of 2000 following the UNHCR guidelines. The per-unit expenditure in keeping the workers in relief shelters was less than keeping them in their own homes. Thus, this option further reduced both the expenditure and the overall economic impact. This can be seen in Figs. 16a, b, 17a, and b. However, relaxing the R value could be detrimental to COVID-19 control and possibly have longer-term impacts. As found in Fig. 18a, and b, the R values could be even double or more than the tolerance limit of 1 in the case of Delhi NCR. That meant every infected person could infect another two people with whom the person came into contact. This would significantly put pressure on the number of hospital admissions which could easily reach the ceiling of the healthcare capacity. Hence, the two key trade-offs for the decision-makers at the governmental level were to decide which one should be allowed to be relaxed, budget or R or both, and the distribution of workers between their own home and relief shelters to control the R vs budget.

**Fig. 19:**
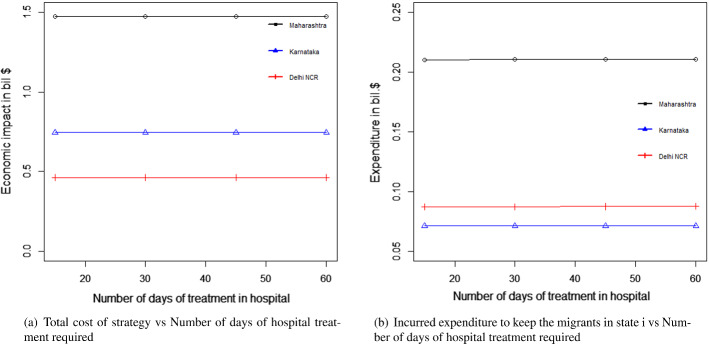
Sensitivity for the number of days a migrant worker requires hospital treatment if infected with COVID19 in state i

We also conducted a sensitivity analysis for the number of days of hospital treatment required if a migrant worker was hospitalized after being infected with COVID-19. The results for the optimal values of the parameters remained the same as shown in Table 6. The rest of the results are presented in Fig. 19a and b. From these figures, the increase in the number of days of hospital treatment has a marginally increasing impact on both the total expenditure and the total impact on the economy for the duration of the planning. When we analyzed the components of the objective function in Eq. (30) and the left-hand side of the constraint (33), we found that the probable number of people infected was a very small part of the total population due to the low probability of infection at the beginning of the pandemic. In consideration, even an increase in the number of days of hospital treatment required would not have had a considerable impact on the total expenditure and total economy. However, in the case of Delhi NCR, the total expenditure was already more than the budget for the base case of 15 days of hospital treatment. Hence, for Delhi NCR, any increase in the number of days of hospital treatment would further violate the budget constraints.

**Fig. 20:**
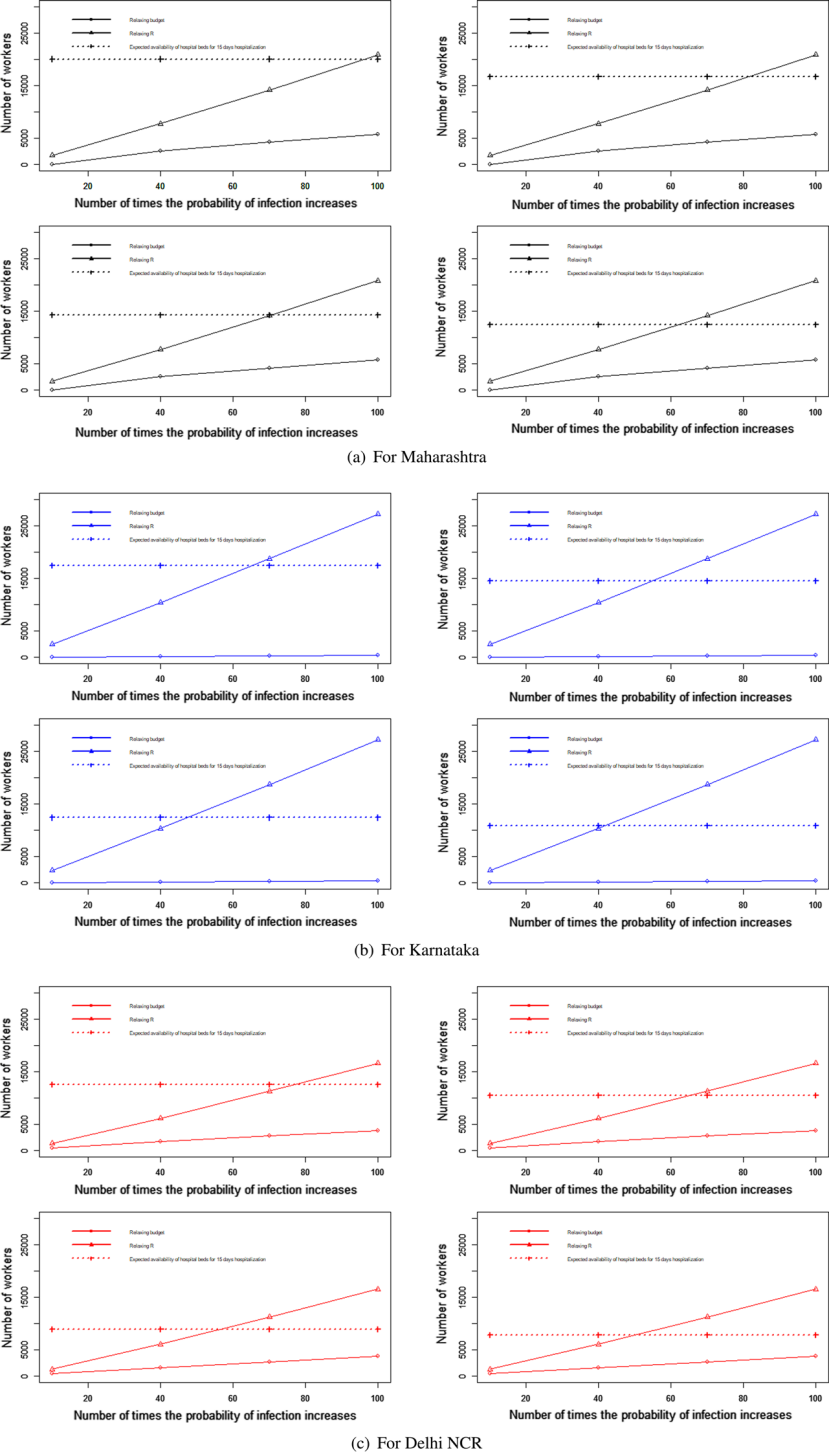
Sensitivity for the number of days required to be treated in hospital by changing the probabilities of infection spread, to keep the migrant workers in the mentioned three states

**Fig. 21:**
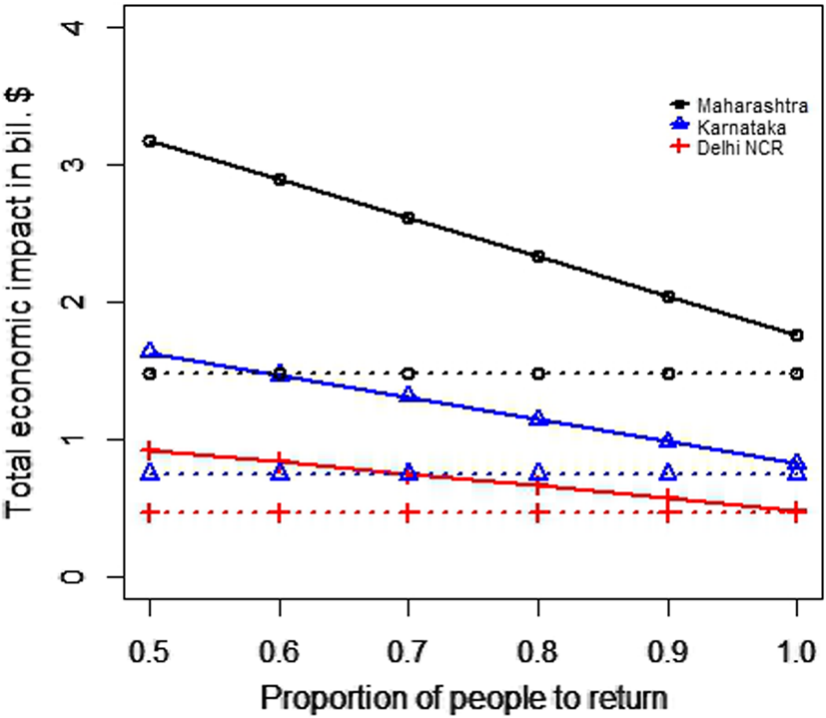
Comparison between two proposed strategies over changing the proportion of returning workers from their home state

We extended the analysis to examine how the increase in the number of days of hospital treatment would have an impact on the model by changing the probability of infection. We derived the results for changing the probability by 10, 40, 70, and 100 times the current probability of infection as suggested by Maji et al. (2020) for 15, 30, 45, and 60 days of hospital treatments respectively. The results are presented in Fig. 20a–c. The solid lines in these figures represent the two cases—relaxing the budget constraints and relaxing the constraints due to R. The dotted lines represented the hospital capacity in each state for various numbers of days of hospital treatment required. Any point on the solid line having a higher vertical intercept than the similar point on the dotted line, meant that for the corresponding case, more people would require hospital treatment than the available hospital capacity. The constraint on hospital treatment was violated in those cases. We found that in cases where the budget constraint could be relaxed, the impact of the increasing number of days of hospital treatment did not violate the corresponding constraint. This can be further explained by the fact that more workers could be allowed to stay in their accommodations rather than giving them shelters in relief camps ($L_{ci}$ decreased with an increase in the probability of infection) where the cost of operation may be lower, but the chance of the spread of the disease is high (Please see Fig. 15a). On the contrary, when we relaxed the constraint on R, there were many cases, (especially for a higher probability of the infection spread), where the hospital occupancy would exceed the weighted hospital capacity, violating the constraint. This would again put pressure on the healthcare system to manage the situation as hospital admission could easily reach its capacity ceiling. Thus, the sensitivity analysis here reiterates the importance of a careful selection of relaxing the constraints from the budget or the R value of the pandemic.

### Comparison between cases

#### Impact of the percentage of workers needing government help and percentage returning to their work state

In the numerical analysis in Sects. 4.4.1 and 4.4.2, we presented two cases: migrant workers being transported to their home state and/or staying in the state where they were working prior to the lockdown. As mentioned in the introduction in Sect. 1, the question is when and which strategy would be appropriate for the state authorities. This section discusses that question.

The results of the two scenarios (4.4.1 and 4.4.2) are compared. We found that keeping migrant workers in the respective states was more economically cost-effective than asking them to travel to their home state. The reason was a COVID-19 outbreak for around 65% of migrant workers requiring government help to either travel to their home state or staying in the state where they were working prior to lockdown. This was found to be true even though the budget constraint for Delhi NCR was violated. This was mainly due to the preventable economic loss from migrant workers not returning to the states they were working after they had traveled to their home state. We found this to be true (by comparing Figs. 10 and 17a) for the cases when the proportion of migrant workers requiring government help increased beyond 65%. We kept the proportion of workers returning to their work state at 45%, assumed for the basic case of migrant workers transported to their home state.

Keeping the proportion of migrant workers requiring government help at 65%, we compared the results of the two proposed cases when the returning proportion of migrant workers at the end of lockdown changed. Again, we found the results favored keeping the migrant workers in state i where they were working before the lockdown (Please see Fig. 21). However, the economic impact was found to be decreasing with the increase in percentage of migrant workers who departed were returning. When 100% of the workers who left were returning, sending the migrant workers home was found to marginally economical in comparison to keeping the migrant workers in their workplace state in the case of Delhi NCR only. This was mainly due to the economic losses that could be prevented when the migrant workers returned and joined the workforce to contribute to that state’s economy.

**Fig. 22:**
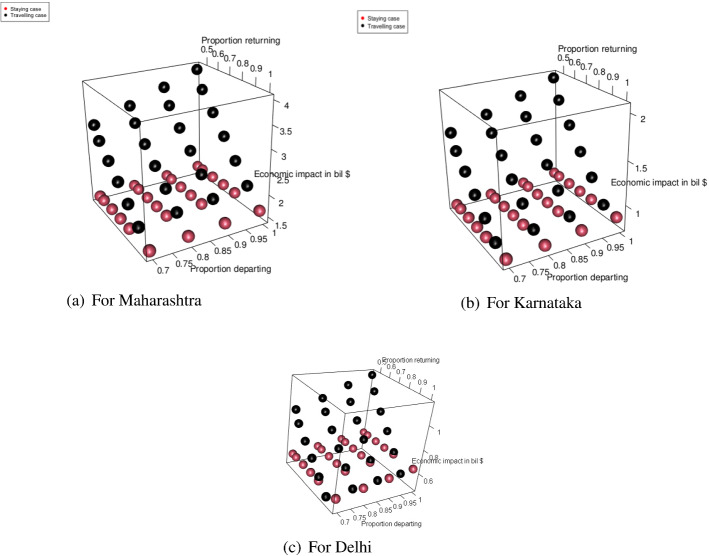
Comparison between two proposed strategies over changing the proportions of both departing and returning workers

We further extended our analysis to compare the results between the two cases by allowing both the proportion of migrant workers leaving their workplace state prior to lockdown and the proportion returning to that state. The results are presented for the three cases separately in Fig. 22a–c. Changing the proportion of migrant workers leaving the state along with changing the proportion returning was not found to have a significantly different impact than what we found from Fig. 21 and discussed above. The monetary impact on the overall economy was found to decrease with any increase in the proportion of returning migrant workers, in the case of migrant workers, sent back to their home state during lockdown. Keeping the migrant workers in their workplace state was found to be mostly economical except in the case of Delhi NCR with 100% workers were returning.

#### Impact of number of days of lockdown

**Fig. 23:**
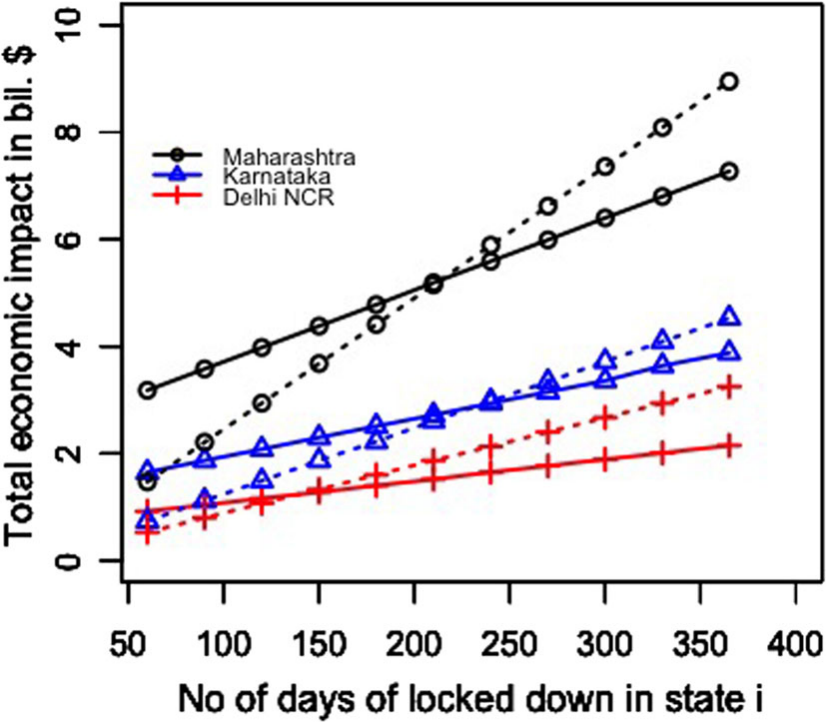
Comparison between two proposed strategies over changing lockdown days

As stated in Sect. 4.1.1, we considered the number of lockdown days to be 60 for the base cases. We compared the overall economic impact of migrant workers being transported interstate (presented in Sect. 4.2) with the cases where migrant workers stayed back in the state where they were working prior to lockdown (presented in 4.3). We found the latter case to be more economical than earlier. However, when the vaccine roll-out was still a work in progress and many countries witnessed the second wave of the virus spread, we extended the analysis for the number of lockdown days to be more than 60. We extended it up to 365 lockdown days which were either full or partial. The results are illustrated in Fig. 23. The bold lines represent the cases of migrant workers transporting interstate and the dotted lines represent the cases where migrant workers staying in state i. As found from Fig. 23, the migrant workers staying in the state where they were working prior to the lockdown was economically beneficial for the states when the number of lockdown days was less. However, things were found to change if the number of lockdown days started to increase. The results were the same for the two cases at an approxiation for all three states: 140 days for Delhi NCR, 214 days in Maharashtra, and 233 days in Karnataka. Any lockdown beyond these many days in each state respectively would favor the economic decision for migrant workers traveling to their home state.

**Fig. 24:**
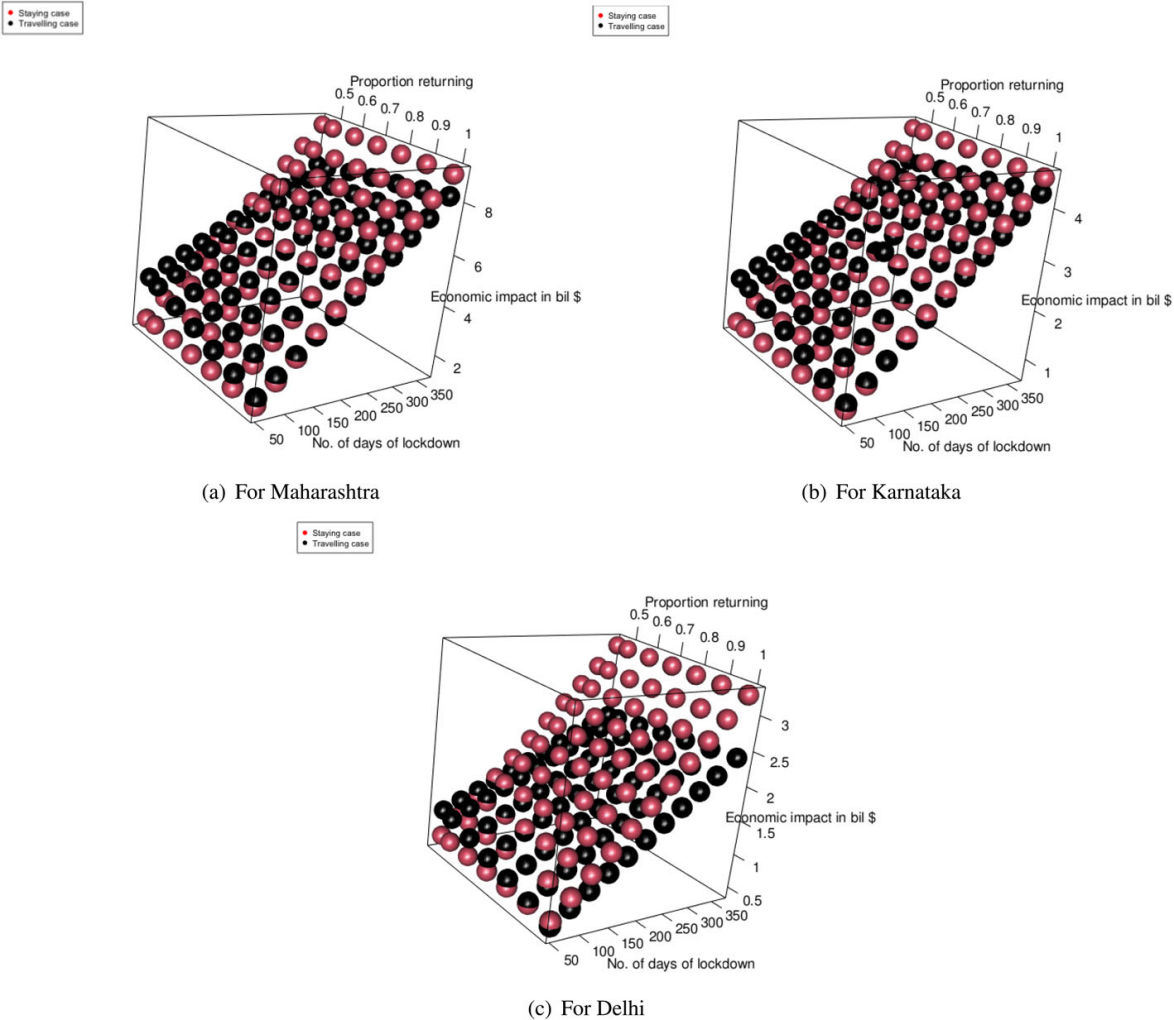
Comparison between two proposed strategies over changing lockdown days and the proportion of returning workers

We further extended our analysis to compare the results between the two cases by allowing both the proportion of migrant workers returning to their workplace state prior to lockdown and the number of days of lockdown to change. The results are presented for the three cases separately in Fig. 24a–c. The change in the number of lockdown days was found to have a significant impact on the overall effect of the two cases. With the increase in the number of lockdown days beyond 60, the strategy of keeping the migrant workers in the state was found to have a greater impact on the overall economy in monetary terms even from a minor increase in the percentage of migrant workers returning from the base value of 45% (As can be seen on the left-hand sides of the Fig. 24a–c). After a certain number of days of lockdown, the strategy of keeping the migrant workers in their workplace states was economically more expensive than sending them to their home state. This was mainly because managing such a large number of migrant workers over a longer period started to have a significant cost impact on the overall economy which was even greater than any economic loss due to migrant workers not returning to their workplace state prior to lockdown.

## Implications of the results

We discussed the implications of the results derived from the numerical experiments (in Sect. 4) in this section. The results of our analysis of the migrant workers travelling case show that the number of migrant workers, who wish to return has a positive impact on the economy. However, the challenging experience of travelling and uncertainty about finding employment on return could discourage the migrant workers who left for their state of domicile to return. Moreover, with the increase in the number of migrant workers leaving, additional facilities needed to be built. These might be restricted due to unavailability or resourcing or budget for the number of medical testing centres which assess migrant workers before they are allowed to board a train. If this constraint is relaxed, an absence of proper testing may raise the risk of the spread of COVID-19 in the states where they are traveling to. We also found that the budget is constrained in the case of Delhi NCR. Delhi NCR which is geographically much smaller than Maharashtra and Karnataka. Thus, any geographically little regions with lower budget allocations may require additional financial support and/or budget allocation for managing the operation of sending the migrant workers back to their state of domicile.

The results from migrant workers staying showed the key trade-off between the budget vs R value of the pandemic. Theoretically, the model constraints of budget and/or R values were violated when certain parameters (the proportion of people requiring government help, and the number of days of hospital admission) were allowed to change. Our results also suggested that allowing the R value to increase to control the budgetary requirements could challenge the healthcare capacity as found in Fig. 18b. Especially with the increasing proportion of workers requiring government assistance and the probability of infection, the health care would exhaust its capacity to accommodate and treat patients infected with COVID-19. This has twofold implications: the possibilities of human casualty and any casualty could translate to 7–8 fold the GDP per capita impact for the Indian economy as indicated by the economic modeling (Goel et al., 2021). This has implications for policy-making purposes in terms of the emergency pandemic budget and its additional allocation to avoid the further spread of the infection, particularly relevant for Delhi NCR where the significant shortfall in the budget had to be covered by the government.

Our analysis suggests that the choice of the right strategy is dependent on variables and parameters of the models including the key factors: the number of migrant workers requiring government assistance, the number of them wishing to return to their workplace state and the number of lockdown days. Theoretically, the results did not offer any unique dominant strategy from the two, but both strategies became dominant at certain times depending on the nature of those parameters and variable values. The results from the analysis show that the proportion of workers willing to return to their work state did not have much significant impact on the results at fewer number of days of lockdown. The results indicated that allowing migrants workers to remain in their work states as economically dominant strategy with 60 days of lockdown except 100% of the departed workers returning at the end of lockdown in the case of Delhi NCR. Fewer lockdown days would result in workers remaining in their work states as the preferred outcome while a higher number of days of lockdown would imply that people traveled to their home state and remained there. However, there was no dominant strategy for longer lockdown periods with an increasing proportion of workers returning. This period was found to be different for different states.

Moreover, our analysis also showed that there is a greater chance of a higher number of migrant workers than the government planned or recommended to coming to train stations. This could lead to more people becoming exposed to the infection. This case might arise due to communication breakdown or even lack of communication due to lack of access to some modern technologies, and uncertainty about the future among many other reasons. We showed that this had significant implications for the R value to become out of control leading to further infection spread and the hospital capacity being exhausted at the same time. Additionally, existing policymaking around the migrant worker movement is diverse among various state-level responses. According to Rajan and Bhagat (2022), labourer is a subject in the ‘concurrent list’ of the Indian constitution. This gives the equal right for states to legislate on matters related to it. This federal nature of India’s response to handling the migrant movement led to the diversity in approaches on the part of different states, leading to further policy-making and practical debate (Rajan & Bhagat, 2022). Thus, the policymakers/decision-makers from the government might require a flexible and alternate approach to address this situation of migrant movement.

Our analysis and results highlight theoretical and methodological implications as well. We developed analytical models to obtain the two different situations of pandemic management corroborated with further numerical analysis: managing the migrant workers traveling inter-state to allow them to escape the repercussions of temporary unemployment and arranging the migrant workers to stay in their workplace states. It has been theorized that any pandemic has the capacity to change not only economic performances, but also can cause demographic shifts, morale shocks and socio-political disturbances. The COVID-19 pandemic should not be only considered from the economic lens of theory, as it has caused high casualties worldwide (Shang et al., 2021). The existing practice of managing the unemployment situation for migrant workers created due to the pandemic was addressed by allowing them to travel to their state of domicile. As noted earlier, certain policy choices such as operating special trains to allow migrant workers to travel were devised (Jain, 2020a). Other policy responses at various state levels included temporary setting up of shelters, providing local bodies with infrastructure for setting up 14-day quarantine centers, setting up government-operated food centers, and offering cash incentives to migrant workers (one of the Indian states Odisha government offered Rs. 1500 or US $20.00 to construction workers registered with the Odisha Building and Construction Workers’ Welfare Board) to register with certain trade bodies for future employment opportunities. However, this strategic policy choice of sending migrant workers to their home has been questioned by many authors on theoretical grounds of labour economics, for example there can be uncertainty for workers returning to their state of domicile who may find it difficult to have agriculture-based jobs that may be available in the longer run (Breman, 2020), whereas some other authors have also admitted the helplessness of the situation. Another debate is the fiscal choices (Thakur & Kumar, 2021) or their limitation (Jose et al., 2020) in the aftermath of the pandemic. On May 13, 2020, the central Government of India announced a stimulus package called “Atmanirbhar Bharat” or “Self-reliant India”. As part of this policy, INR of 2 trillion (appx US $300 billion) would be distributed to the states for migrant welfare under the Prime Minister’s Citizen Assistance and Relief in Emergency Situations (PM-CARES) (Rajan, 2020; Rajan & Bhagat, 2022). However, the authors have debated the effectiveness of these stimulus packages due to various complexities involved. Without participating in these debates, our proposed models theoretically highlights a possible alternative strategy of managing this migrant worker movement for consideration. Furthermore, our analysis offers flexibility to compare different strategies (sending migrant workers versus allowing them to settle within their workplace states before COVID-19) and select the optimal one in different situations. This is a theoretical attempt at being more flexible and composite while addressing the labour migration issues as noted by Breman (2020). One of the proposed sub-strategic choices (allowing migrant workers to stay in their homes in their workplace states) requires stimulus help from governmental bodies. Many notable economists questioned similar fiscal stimulus packages about their functionality in a monetarist economy, but the authors Thakur and Kumar (2021) concluded similar fiscal expansion would be the only way out due to the severity of the situation. Our models offer the flexibility of a strategic choice without any monotonous dominance of strategy over another which can offer a more practical solution.

## Conclusion

We started this research with the overarching aim to determine the right strategy of sending the migrant workers back to their state of domicile or making arrangements in the states where the migrant workers were working before the lockdown. We proposed analytical models to obtain the two situations and corroborated that with further numerical analysis. Our analysis enlightens the different strategic choices for managing the migrant workers’ movement (Allowing them to travel to their state of domicile or allowing them to stay). Our analysis also summarizes the fact that the right strategy is dependent on variables and parameters of the models including the key factors: the number of migrant workers requiring government assistance, the number of them who wished to return to the states where they had been working before the lockdown and the number of days of lockdown. There is no unique dominant strategy among the two but both strategies became dominant at certain times depending on the nature of those parameter and variable values. Thus, the policymakers and decision-makers from the government might require a flexible and holistic approach to address this situation of migrant movement. We also found the need for an additional emergency pandemic budget and its allocation to avoid the further spread of the infection in certain situations as discussed in Sects. 4 and 5. This has further policy implications in terms of budgetary allocation trade-offs among various activities that fall under the government budget for future pandemic preparation. In addition, we found situations where the existing infrastructural facilities such as medical testing centres, and hospitals could reach capacity constraints. In those situations, a further private-public partnerships would be required.

The findings of the current study address the research gap in offering analytical approaches that can be used for addressing the trade-off between the economic impact against the epidemiological requirements of the COVID-19 virus spread in an emerging economy like India where there are several economic constraints which may potentially restrict any pandemic management. The current research was based on a specific migrant worker movement during COVID-19 in India. The world including Indian society which has been recovering in the positive direction.

However, the implications are far-reaching for future research in the areas of humanitarian logistical issues and subsequent policy making validates the importance of the current research. Anecdotal evidence supports similar migrant movement occurrences which led to similar disastrous situations, for example, a break out of pneumonic plague in Surat, Gujrat in India in August 1994 caused widespread panic. This outbreak led to more than 200,000 people fleeing the city (Leo, 2020). During the 2003 SARS outbreak, a significant number of citizen left Beijing. More recent evidence suggested an exodus of people in Italy to avoid imposition of red zones and other mobility restrictions during COVID19 (Giuffrida & Tondo, 2020). Thus, preparedness with alternative strategic choices will be pivotal for better management of similar situations of any future pandemic.

As demonstrated earlier, the entire research was conducted based on the published datasets from: various governmental and non-governmental sources, news media, and peer-reviewed journal articles (where the authors used and noted the relevant datasets). These helped to generate the relevant results as discussed in Sect. 4. Thus, the approach discussed in this research will be helpful for any future similar humanitarian disaster management scenario where the decision-makers may not be able to gather data from primary sources due to the emergent and sensitive nature of the situation.

Summarizing the discussion of the last two paragraphs, we note the contributions of the current research as

- Modeling a realistic policy decision regarding the transportation of migrant workers in India while considering both economic and infection risks using optimization techniques.
- Demonstrating the usefulness of data-driven decision-making by collecting relevant data which governments can use to take such decisions.
- Analyzing trade-offs and developing recommendations based on threshold values of the percentage of workers willing to return and the days of lockdown.

The research has certain limitations which stem from the assumptions, for example, it did not consider the economic impact in the home state of the migrant workers when they travel to those states and how the local governments in those states manage their social well-being as well as keeping the rate of infections under control. Furthermore, our model did not consider any asymptomatic infection in the calculations for either of the two main cases. It would be interesting to see, how the inclusion of these additional parameters impacts the results in future research work. In the case of the migrant workers staying in the work state, the proposed model did not consider any resettlement money for those who were staying in relief shelters. This can be included in future work using the UNHCR guidelines. There are also opportunities to extend this research as multi-period models considering different phases of lockdown restrictions. Such multi-period models can also incorporate stochasticity of the proportion of migrant workers moving from their state of work to their state of domicile as well as the proportion of such migrant workers returning to their work state.

## Data Availability

The research was conducted based on the secondary data gathered from various publicly available governmental and non-governmental sources, and in some cases, we assumed data points based on a realistic estimation inferred from the collected secondary data. All the sources have been duly referenced following the standard referencing conventions. No primary data have been collected based on human and/or animal participation.

## Footnotes

1 Although, year 2021 is the current census, but the data was not not available when the research was conducted. Please see https://censusindia.gov.in/2021-Schedules/index.html.
